# AP-2 Adaptor Complex-Dependent Enhancement of HIV-1 Replication by Nef in the Absence of the Nef/AP-2 Targets SERINC5 and CD4

**DOI:** 10.1128/mbio.03382-22

**Published:** 2023-01-09

**Authors:** Balaji Olety, Yoshiko Usami, Yuanfei Wu, Paul Peters, Heinrich Göttlinger

**Affiliations:** a Department of Molecular, Cell and Cancer Biology, University of Massachusetts Chan Medical School, Worcester, Massachusetts, USA; Columbia University Medical Center

**Keywords:** AP-2, CD4, LCK, Nef, PAK2, SERINC5, human immunodeficiency virus, virus replication

## Abstract

Human immunodeficiency virus type 1 (HIV-1) Nef hijacks the clathrin adaptor complex 2 (AP-2) to downregulate the viral receptor CD4 and the antiviral multipass transmembrane proteins SERINC3 and SERINC5, which inhibit the infectivity of progeny virions when incorporated. In Jurkat Tag T lymphoid cells lacking SERINC3 and SERINC5, Nef is no longer required for full progeny virus infectivity and for efficient viral replication. However, in MOLT-3 T lymphoid cells, HIV-1 replication remains highly dependent on Nef even in the absence of SERINC3 and SERINC5. Using a knockout (KO) approach, we now show that the Nef-mediated enhancement of HIV-1 replication in MOLT-3 cells does not depend on the Nef-interacting kinases LCK and PAK2. Furthermore, Nef substantially enhanced HIV-1 replication even in triple-KO MOLT-3 cells that simultaneously lacked the three Nef/AP-2 targets, SERINC3, SERINC5, and CD4, and were reconstituted with a Nef-resistant CD4 to permit HIV-1 entry. Nevertheless, the ability of Nef mutants to promote HIV-1 replication in the triple-KO cells correlated strictly with the ability to bind AP-2. In addition, knockdown and reconstitution experiments confirmed the involvement of AP-2. These observations raise the possibility that MOLT-3 cells express a novel antiviral factor that is downregulated by Nef in an AP-2-dependent manner.

## INTRODUCTION

Nef is a virulence factor of human immunodeficiency virus type 1 (HIV-1) and other primate lentiviruses that is required for high virus loads and for viral pathogenicity *in vivo* (1, 2). Although Nef is not an essential HIV-1 gene product, it can substantially enhance HIV-1 spreading among primary human CD4^+^ T cells (3, 4). Notably, Nef proteins from all three HIV-1 groups and from several highly divergent simian immunodeficiency viruses (SIVs) efficiently promote HIV-1 replication in human peripheral blood mononuclear cells (PBMC), indicating that the enhancement of virus spreading is a highly conserved function of Nef (5). However, the underlying mechanism remains to be fully elucidated.

Among the many reported *in vitro* activities of Nef, one of the most conserved is the downregulation of the viral entry receptor CD4 from the surface of infected cells (6–8). This activity involves the formation of a ternary complex between Nef, the cytoplasmic domain of CD4, and the clathrin adaptor complex 2 (AP-2), which orchestrates the internalization of CD4 through clathrin-mediated endocytosis (9, 10). Additionally, Nef engages AP-2 to prevent the incorporation of the antiviral multipass transmembrane proteins SERINC3 and SERINC5 into progeny virions (11–15). Nef also co-opts the AP-1 clathrin adaptor complex to downregulate certain major histocompatibility complex class I (MHC-I) molecules (16).

The Nef-mediated downregulation of CD4 and MHC-I is thought to protect infected cells from different forms of cell-mediated cytotoxicity (17–19). However, it has also been shown that high levels of cell surface CD4 impair the release and infectivity of HIV-1 virions and that Nef counteracts these effects by downregulating CD4 (20, 21). Furthermore, it has been observed that the ability of Nef to downregulate CD4 strongly correlates with its ability to enhance HIV-1 replication in primary cells *in vitro* (22). Consistent with these *in vitro* results, the ability to downregulate CD4 correlated with the Nef-mediated enhancement of HIV-1 pathogenicity in humanized mouse models (23, 24). Together, these findings suggest that the downregulation of CD4 by Nef can directly promote HIV-1 replication.

The downregulation of SERINC5 by Nef largely accounts for its well-documented ability to enhance the specific infectivity of progeny virions (11, 12, 25). Although most HIV-1 Nef proteins additionally downregulate SERINC3, exogenous SERINC3 has little effect on HIV-1 progeny virus infectivity, whereas exogenous SERINC5 can lower it dramatically (26). Nevertheless, the downregulation of endogenous SERINC3 likely contributes to the effect of Nef on progeny virus infectivity (12). Furthermore, at least in Jurkat Tag cells, the downregulation of SERINC3 and SERINC5 accounts for the Nef-mediated enhancement of HIV-1 spreading (12). SERINC3 and SERINC5 have also been shown to inhibit HIV-1 by promoting innate immune signaling (27). Consistent with these observations, the selective disruption of the SERINC5 antagonism of a simian immunodeficiency virus (SIV) Nef protein impaired virus replication in primary CD4^+^ T cells, attesting to the biological relevance of this function of Nef (28).

In addition to cellular trafficking machinery, Nef engages host proteins to modulate the activation state and the actin cytoskeleton of infected cells (29). In particular, it has long been known that Nef interacts with the SH3 domains of certain Src family tyrosine kinases through a conserved proline-rich motif (30, 31). In an early study, Nef interacted only with the Src kinases HCK and LYN, which are both expressed at rather low levels in CD4^+^ T cells (30). However, inhibitors of Nef-dependent HCK activation have been shown to block HIV-1 replication in macrophages, which express substantial amounts of HCK (32). It subsequently emerged that Nef also interacts with the Src kinase LCK, which is highly expressed in primary CD4^+^ T cells and plays a key role in T cell receptor signaling (33–35). Notably, Nef reroutes LCK from the plasma membrane to an intracellular compartment (36), and triggers LCK-dependent signaling at the trans-Golgi network (37), which has been suggested to facilitate HIV-1 replication (37).

Another well-documented interaction partner of Nef is the p21-activated kinase 2 (PAK2) (38, 39), a member of the highly conserved PAK family of serine/threonine protein kinases that regulate the cytoskeleton and function as effectors of the RHO family GTPases CDC42 and RAC (40). The ability of Nef to interact with PAK2 is generally conserved (41) as is the ability of Nef to trigger actin cytoskeleton remodeling in a PAK2-dependent manner (42). Furthermore, the ability of Nef to associate with activated PAK2 has been implicated in its ability to stimulate HIV-1 replication in freshly isolated primary T cells (43).

Nef enhances HIV-1 replication in some T cell lines, but the effects can be rather modest (44). However, we recently observed that HIV-1 replication in MOLT-3 T lymphoid cells is highly dependent on Nef (26). Our results also indicated that unlike in Jurkat Tag cells, the ability of Nef to antagonize SERINCs did not account for its effect on HIV-1 replication in MOLT-3 cells or in primary cells (26). Moreover, Nef robustly promoted HIV-1 replication in MOLT-3 cells that predominantly expressed a Nef-resistant CD4 molecule, indicating that CD4 downregulation was not required (26). We now show that the Nef-interacting kinases LCK and PAK2 are also not required. Furthermore, Nef remained fully capable of promoting HIV-1 spreading even in triple-knockout (KO) MOLT-3 cells that simultaneously lacked the Nef/AP-2 complex targets SERINC3, SERINC5, and CD4 and expressed only a Nef-resistant CD4 to support HIV-1 entry. Nevertheless, our results indicate that AP-2 remained specifically required for the enhancement of HIV-1 replication by Nef in these cells. These observations point to the existence of an unknown antiviral factor that is counteracted by Nef in an AP-2-dependent manner.

## RESULTS

### Propagation of SERINC-resistant HIV-1 in primary cells remains Nef dependent.

**FIG 1:**
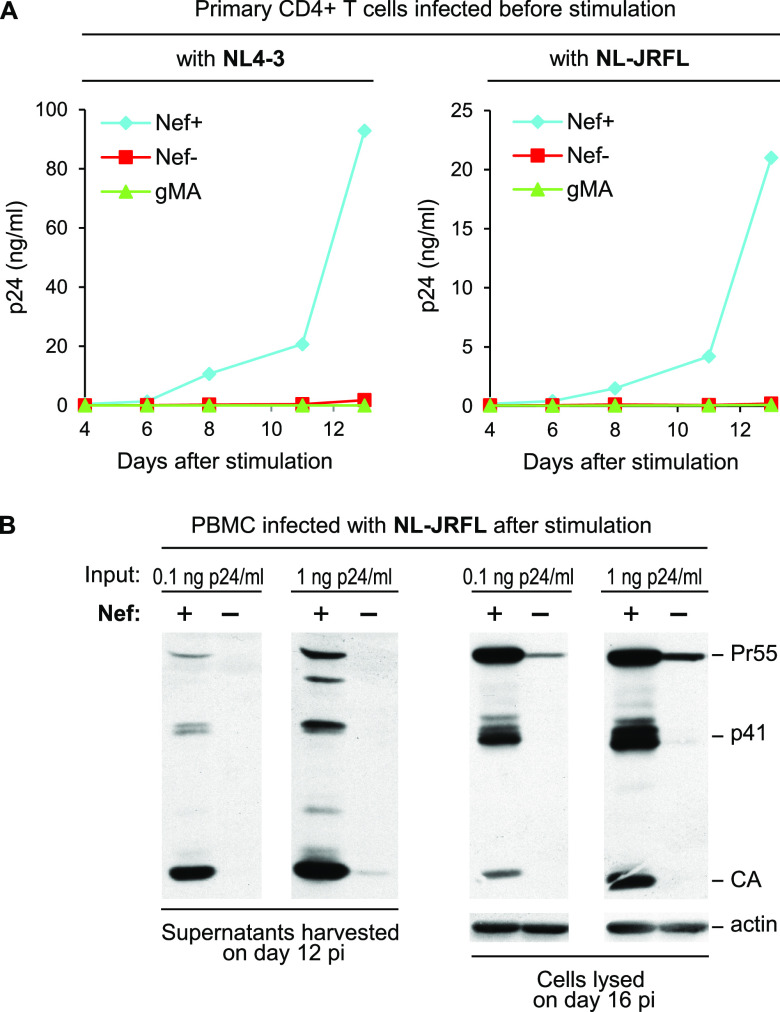
Propagation of SERINC-resistant HIV-1 in primary cells remains Nef-dependent. (A) Virus growth curves showing that Nef enhances the replication of both SERINC-sensitive (NL4-3) and SERINC-resistant (NL-JRFL) HIV-1 strains in primary human CD4^+^ T cells infected prior to stimulation. In contrast, the potent SERINC antagonist glycoMA (gMA) does not enhance HIV-1 replication in this context. CD4^+^ T cells were enriched by negative selection, infected with HIV-1_NL4-3_ or HIV-1_NL-JRFL_ (at 1 ng p24/mL), and stimulated with PHA on day 3 after infection. HIV-1 replication was examined by monitoring p24 accumulation in the culture supernatants over time. (B) Nef enhances the spreading of the SERINC-resistant NL-JRFL strain in prestimulated human PBMC. After stimulation with PHA, PBMC were infected with 0.1 or 1 ng p24/mL, and culture supernatants and cell lysates were examined by Western blotting with anti-CA on day 12 and day 16 postinfection, respectively.

We previously reported that the SERINC antagonist glycoMA, when inserted into HIV-1_NL4-3_ in place of *nef*, enhanced HIV-1 propagation in Jurkat cells to a comparable extent as Nef itself. In marked contrast, glycoMA had no effect on the spreading of Nef^−^ HIV-1_NL4-3_ in MOLT-3 cells or in primary human PBMC. To confirm that glycoMA cannot substitute for Nef in primary human cells, we have now examined its ability to promote the spreading of HIV-1_NL4-3_ in CD4^+^ T cells. Primary human CD4^+^ T cells were infected immediately after purification by negative selection and stimulated with phytohemagglutinin (PHA) 3 days later, conditions under which robust effects of Nef on HIV-1 spreading have been observed. As shown in Fig. 1A, Nef was critical for the propagation of HIV-1_NL4-3_ in primary CD4^+^ T cells under these conditions, and glycoMA did not rescue the replication defect of Nef^−^ HIV-1_NL4-3_. Since glycoMA potently counteracts both SERINC3 and SERINC5, these results implied that the considerable replication defect of Nef^−^ HIV-1_NL4-3_ in primary CD4^+^ T cells was not solely due to an inability to counteract SERINCs.

To further examine this issue, we used a variant of HIV-1_NL4-3_ termed HIV-1_NL-JRFL_, which has the *env* gene replaced with that of the primary HIV-1 strain JRFL. Importantly, unlike the Env protein of HIV-1_NL4-3_, which is highly SERINC5 sensitive, Env_JRFL_ is largely resistant to endogenous levels of SERINC5. Nevertheless, the propagation of HIV-1_NL-JRFL_ in primary CD4^+^ T cells that were infected prior to stimulation was similarly dependent on Nef as the propagation of HIV-1_NL4-3_, and neither virus was rescued by glycoMA (Fig. 1A). In a separate experiment, we observed that Nef also substantially enhanced the spread of HIV-1_NL-JRFL_ in primary human PBMC that were infected after PHA stimulation (Fig. 1B). Taken together, these observations provide evidence for a SERINC-independent restriction of Nef^−^ HIV-1 in primary CD4^+^ T cells.

### Nef strongly enhances HIV-1 replication in cells lacking LCK.

Like in primary CD4^+^ T cells, the replication of SERINC-resistant HIV-1 in MOLT-3 T lymphoid cells depends on Nef (45). Indeed, MOLT-3 cells severely restrict the replication of Nef^−^ HIV-1 even in the absence of SERINC3 and SERINC5 (45). Therefore, we used MOLT-3 cells as a model system to examine the involvement of other host factors. One factor that has been implicated in the enhancement of HIV-1 replication by Nef in T cells is the lymphocyte-specific tyrosine kinase LCK (37, 46). Several studies indicate that Nef physically interacts with LCK and affects LCK-mediated signaling (33–35). However, whether Nef has a positive or negative effect on LCK has been controversial. There is evidence that Nef activates LCK and that this ultimately leads to the induction of HIV-1 transcription and replication (47). In contrast, other studies conclude that Nef impairs the kinase activity of LCK (33, 34), which could conceivably support virus replication by inhibiting activation-induced cell death (29). It has also been proposed that Nef does not affect the overall activity of LCK but rather triggers the relocalization of active LCK from the plasma membrane to intracellular membranes (37).

**FIG 2:**
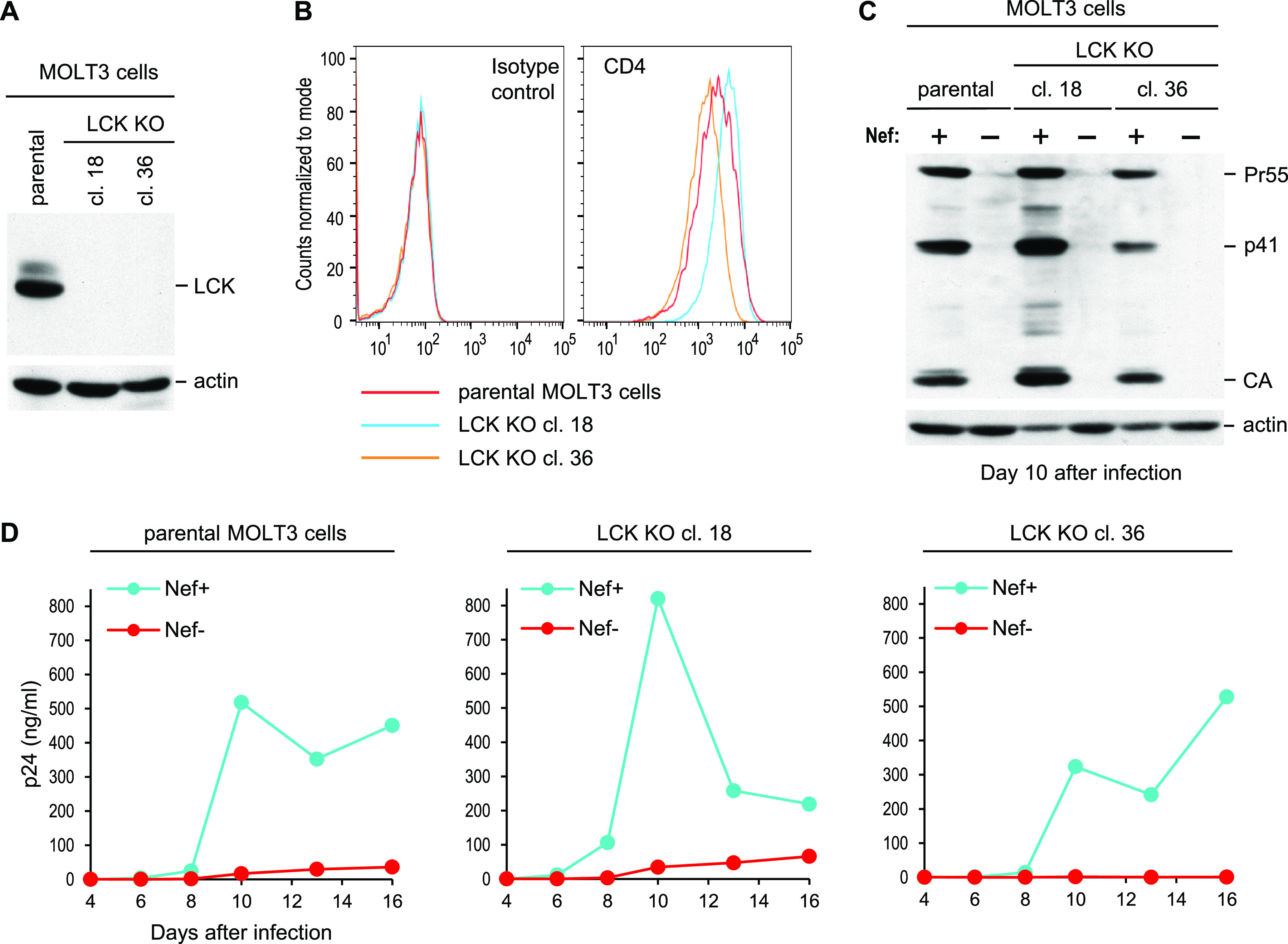
Nef strongly enhances HIV-1 replication in MOLT-3 cells lacking LCK. (A) LCK expression in parental MOLT-3 cells and in LCK KO clones analyzed by Western blotting. (B) CD4 surface levels on the same cells analyzed by flow cytometry. (C) Western blots showing the effects of Nef on HIV-1 replication in parental MOLT-3 cells and in LCK KO clones. The cells were infected with equal amounts (0.2 ng p24/mL) of Nef^+^ or Nef^−^ HIV-1_NL4-3_, and cell lysates were examined with anti-CA and anti-actin 10 days after infection. (D) Virus replication in the same cultures monitored by measuring p24 accumulation in the supernatants over time.

Using CRISPR/Cas9-mediated gene editing, we obtained two MOLT-3-derived clones that completely lack LCK (Fig. 2A) but exhibit CD4 surface levels comparable to those on the parental MOLT-3 cells (Fig. 2B), even though LCK is normally tightly associated with the CD4 cytoplasmic domain and can affect the internalization rate of CD4 (48). In both LCK KO clones, the ability of HIV-1_NL4-3_ to replicate remained as dependent on Nef as in parental (LCK^+^) MOLT-3 cells, as determined by measuring Gag expression in the infected cells by Western blotting (Fig. 2C) and by monitoring virus release over time in two independent experiments (Fig. 2D; see also Fig. S1 in the supplemental material). These results demonstrate that LCK is dispensable for the marked enhancement of HIV-1 replication by Nef in MOLT-3 cells.

10.1128/mbio.03382-22.1 FIG S1 Nef strongly enhances HIV-1 replication in MOLT-3 cells lacking LCK. In this repeat experiment, parental MOLT-3 cells and LCK KO clones were infected with Nef^+^ or Nef^−^ HIV-1_NL4-3_ as described in Fig. 2, and virus replication was monitored by measuring p24 accumulation in the supernatants over time. Download FIG S1, PDF file, 0.05 MB. Copyright © 2023 Olety et al. 2023 Olety et al. https://creativecommons.org/licenses/by/4.0/ This content is distributed under the terms of the Creative Commons Attribution 4.0 International license.

### Nef can fully support HIV-1 replication in the absence of all group I PAKs.

Early studies have revealed that a Nef-associated kinase (NAK) is a member of the p21-activated kinase (PAK) family of serine/threonine kinases (38, 49, 50). NAK was later identified as PAK2 (39, 51), and several lines of evidence indicate that the Nef-PAK2 interaction plays an important role in HIV-1 replication by affecting the apoptosis of infected cells (52), their activation state (43, 53), or their ability to transmit HIV-1 via cell-cell contacts (54).

**FIG 3:**
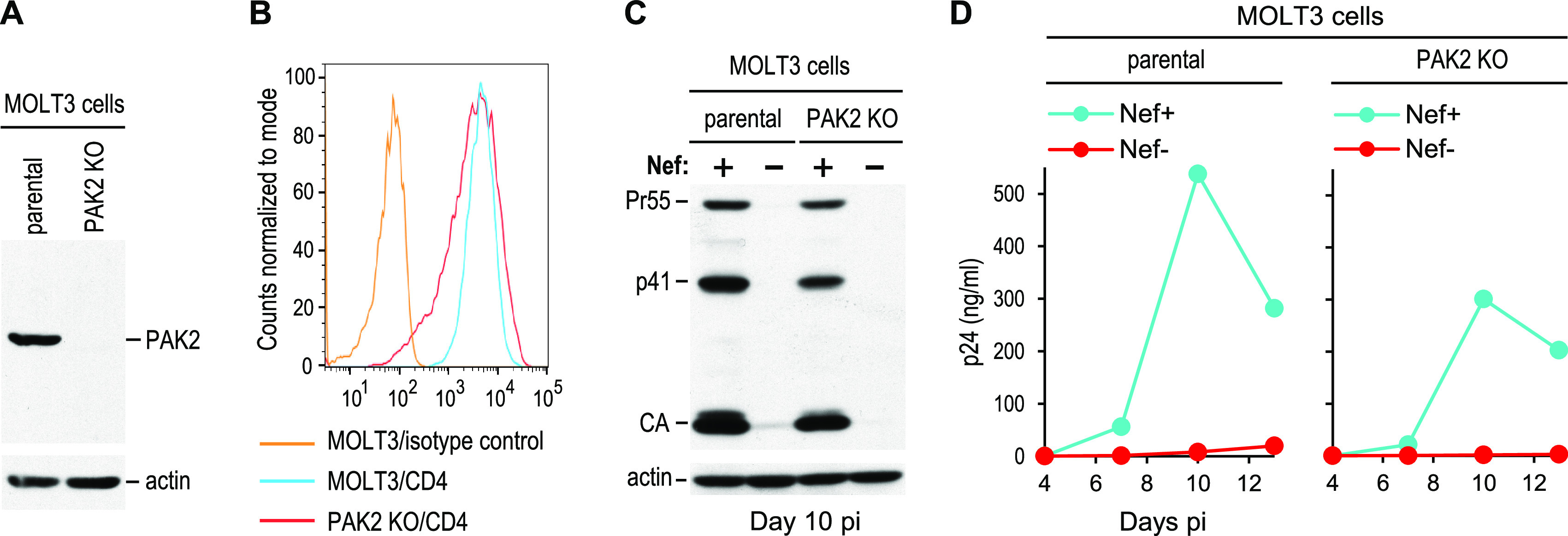
Nef can strongly enhance HIV-1 replication in cells lacking PAK2. (A) PAK2 expression in parental and in PAK2 KO MOLT-3 cells analyzed by Western blotting. (B) CD4 surface levels on the same cells analyzed by flow cytometry. (C) Western blots showing that the effects of Nef on HIV-1 replication in parental and in PAK2 KO MOLT-3 cells are comparable. The cells were infected with 0.2 ng p24/mL of Nef^+^ or Nef^−^ HIV-1_NL4-3_, and cell lysates were analyzed as in Fig. 2C. (D) Virus replication monitored in parallel by measuring p24 accumulation in the supernatants.

To examine whether PAK2 is strictly necessary for the ability of Nef to enhance HIV-1 replication, we used the CRISPR/Cas9 approach to obtain PAK2 KO MOLT-3 cells. While PAK2 was readily detectable by Western blotting in the parental MOLT-3 cells, PAK2 could not be detected in the PAK2 KO MOLT-3 cells (Fig. 3A). However, the levels of surface CD4 on the parental and PAK2 KO MOLT-3 cells were comparable (Fig. 3B). Overall, the cells lacking PAK2 were somewhat less permissive for HIV-1 replication than the parental cells (Fig. 3C and D). However, in two independent experiments, Nef remained clearly capable of enhancing HIV-1 replication in the absence of PAK2 (Fig. 3C and D; see also Fig. S2 in the supplemental material).

**FIG 4:**
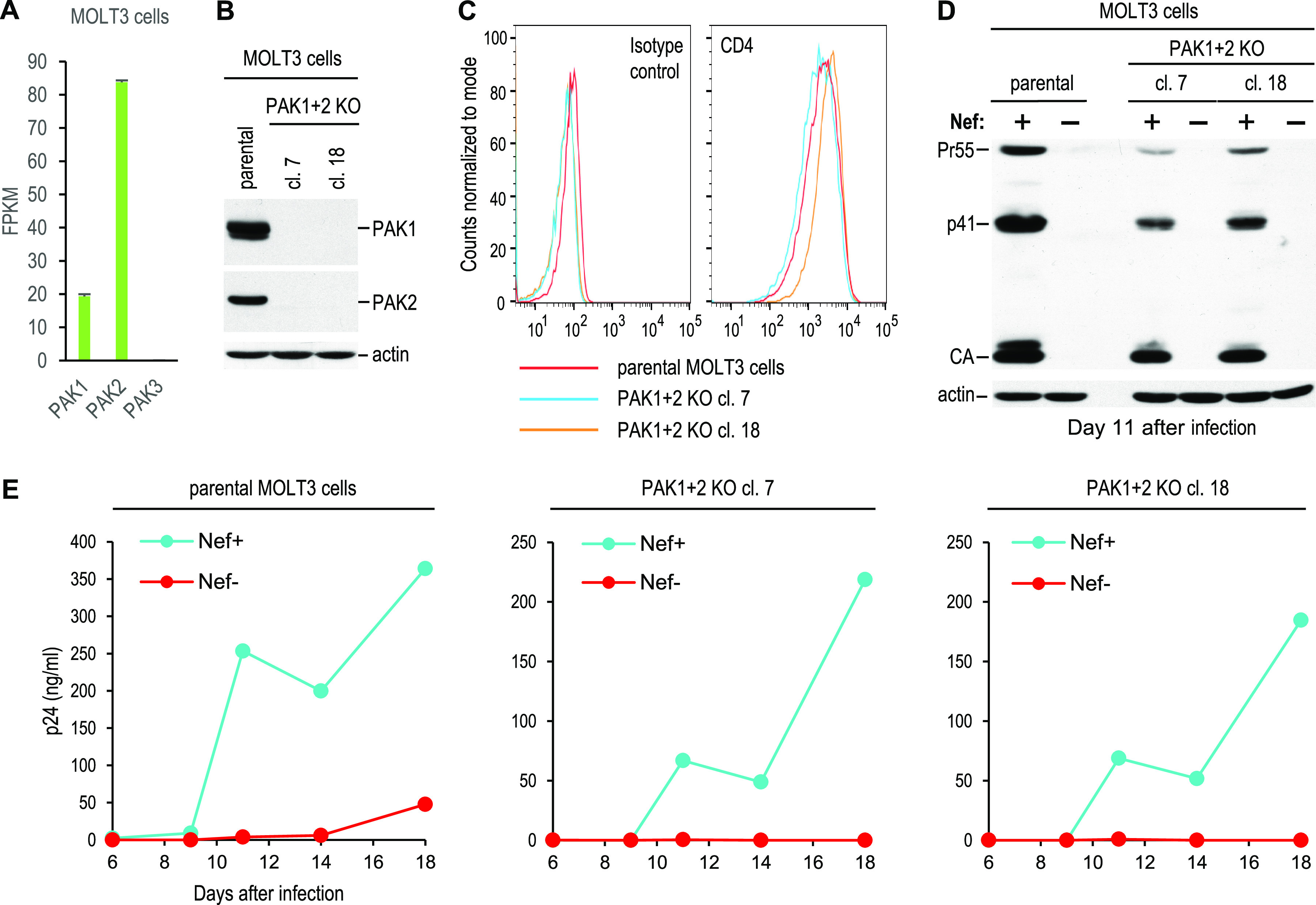
Nef can fully support HIV-1 replication in the absence of all group I PAKs. (A) Expression of group I PAK mRNAs in MOLT-3 cells quantified by transcriptome sequencing (RNA-seq) as fragments per kilobase of transcript per million mapped reads (FPKM). (B) Expression of PAK1 and PAK2 in parental MOLT-3 cells and in PAK1/PAK2 double-KO clones analyzed by Western blotting. (C) CD4 surface levels on the same cells analyzed by flow cytometry. (D and E) Replication of Nef^+^ and Nef^−^ HIV-1_NL4-3_ in the same cells after infection with 0.2 ng/mL p24, monitored by Western blotting of cell lysates with anti-CA (D) and by measuring p24 accumulation in the supernatants (E).

10.1128/mbio.03382-22.2 FIG S2 Nef can strongly enhance HIV-1 replication in the absence of all group I PAKs. In these repeat experiments, the ability of Nef to support HIV-1 replication in MOLT-3 cells lacking either PAK2 alone or both PAK1 and PAK2 (and thus all group I PAKs) was examined as described in Fig. 3 and Fig. 4. Download FIG S2, PDF file, 0.05 MB. Copyright © 2023 Olety et al. 2023 Olety et al. https://creativecommons.org/licenses/by/4.0/ This content is distributed under the terms of the Creative Commons Attribution 4.0 International license.

Although most studies suggest that NAK is PAK2, it has also been reported that Nef can bind to and activate PAK1 (55), which together with PAK2 and PAK3 belongs to the group I PAKs (40). Furthermore, it has been shown that the depletion of PAK1, and to a lesser extent of PAK3, blocks HIV-1 infection in Jurkat cells (56). We, therefore, examined the possibility that, in the absence of PAK2, Nef can use other group I PAKs to enhance HIV-1 replication.

As shown in Fig. 4A, MOLT-3 cells express both PAK1 and PAK2 at the mRNA level but lack mRNA encoding PAK3. Thus, to obtain cells that lack all group I PAKs, PAK2 KO MOLT-3 cells were subjected to a second round of gene editing with a single guide RNA (sgRNA) targeting PAK1. Two clones that lack both PAK1 and PAK2 (Fig. 4B) but exhibit surface CD4 levels comparable to the parental cells (Fig. 4C) were infected with Nef^+^ or Nef^−^ HIV-1_NL4-3_, and virus replication was examined by monitoring Gag expression levels (Fig. 4D) and the release of p24 antigen (Fig. 4E). Although both double-KO clones proved to be less permissive than the parental cells, Nef remained fully capable of enhancing HIV-1 replication in both clones (Fig. 4D and E). Furthermore, the enhancement of HIV-1 replication by Nef in the double-KO clones was reproducible in a repeat experiment (Fig. S2). We conclude that, at least in MOLT-3 cells, group I PAKs are not required for this activity of Nef.

### Nef requirement in cells that lack the Nef/AP-2 targets SERINC3, SERINC5, and CD4.

We previously showed that Nef profoundly enhanced HIV-1 replication in MOLT-3 cells that ectopically expressed an excess amount of a Nef-resistant CD4 molecule (45). However, because of the presence of both Nef-resistant ectopic CD4 and of endogenous CD4 in these cells, we could not strictly rule out that the downregulation of the Nef-sensitive endogenous CD4 played a role in the enhancement of HIV-1 replication.

**FIG 5:**
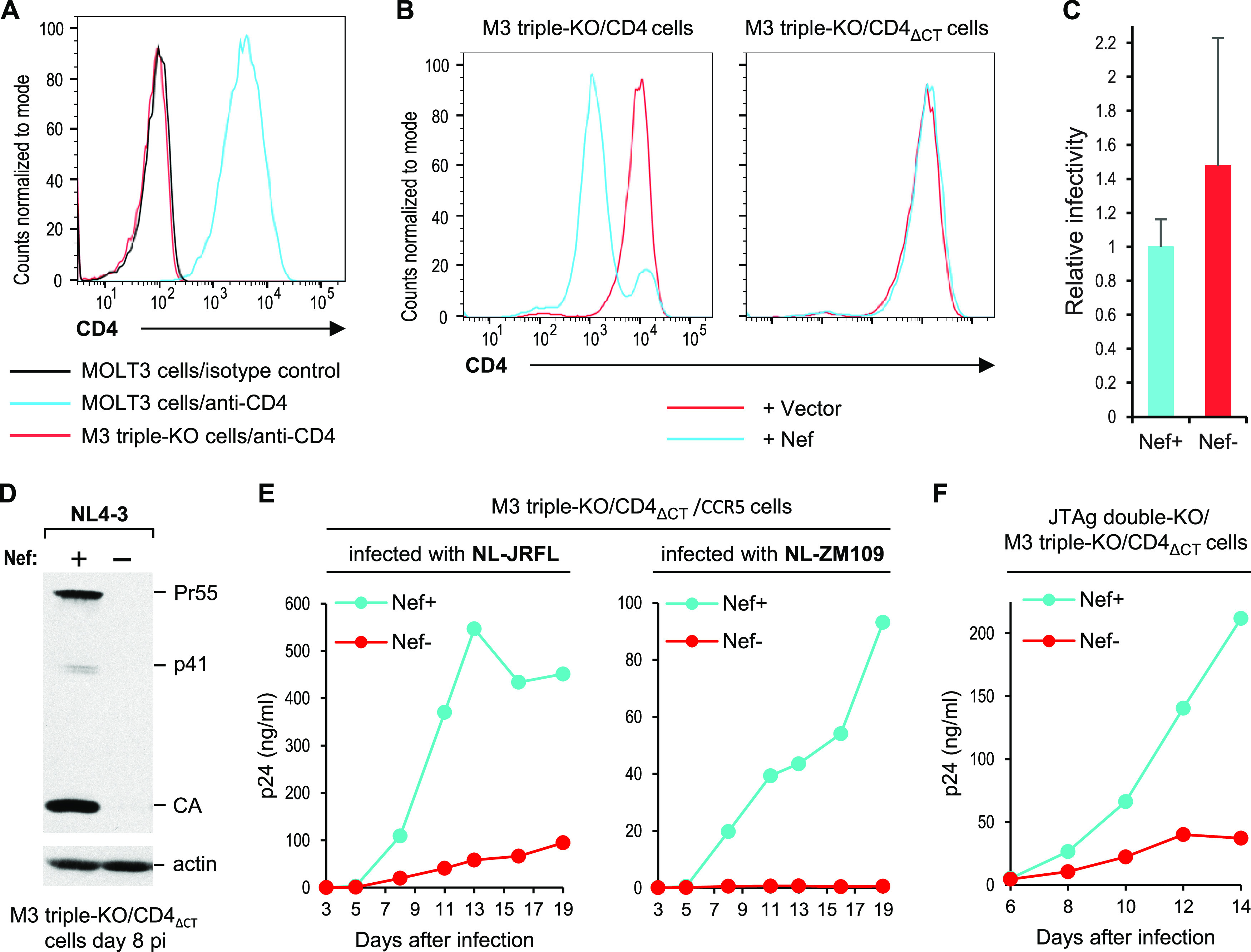
Nef remains required for efficient HIV-1 replication in MOLT-3 cells lacking SERINCs and Nef-sensitive CD4. (A) CD4 surface levels on parental MOLT-3 cells and on M3 triple-KO (SERINC3/SERINC5/CD4 KO) cells analyzed by flow cytometry. (B) CD4 surface levels on M3-triple-KO/CD4 and M3 triple-KO/CD4_ΔCT_ cells stably transduced with empty MSCVpuro (vector) or with a version expressing Nef_LAI_. (C) Relative infectivities of Nef^+^ and Nef^−^ HIV-1_NL4-3_ virions produced in M3 triple-KO/CD4_ΔCT_ cells measured using MOLT-3/ZsGreen reporter cells. Data are mean of three experiments with SD. (D) Replication of Nef^+^ and Nef^−^ HIV-1_NL4-3_ in M3 triple-KO/CD4_ΔCT_ cells. The cells were infected with 0.2 ng p24/mL, and cell lysates were examined by Western blotting with anti-CA and anti-actin on day 8 postinfection (p.i.). (E) Replication of Nef^+^ and Nef^−^ versions of the R5-tropic NL-JRFL and NL-ZM109 viruses in M3 triple-KO/CD4_ΔCT_/CCR5 cells infected with 0.2 ng p24/mL, monitored by measuring p24 accumulation in the supernatants. (F) Replication of Nef^+^ and Nef^−^ HIV-1_NL4-3_ in heterokaryons formed between M3 triple-KO/CD4_ΔCT_(GFP_8-11_) cells and JTAg double-KO(GFP_1-7_) cells transiently expressing the HN and F proteins of NDV. GFP-positive cells were sorted by FACS and infected with 5 ng p24/mL. Virus replication was monitored by measuring p24 accumulation in the supernatants.

To address this possibility, we first knocked out endogenous CD4 and then ectopically expressed a Nef-resistant CD4 in the KO cells. As parental cells for the CD4 knockout, we used previously generated double-KO MOLT-3 cells lacking SERINC3 and SERINC5, in which HIV-1 replication remains highly dependent on Nef (45). In addition to SERINC3 and SERINC5, the resulting MOLT-3 (M3) triple-KO cells completely lack CD4 (Fig. 5A).

CD4 expression was restored after the stable transduction of M3 triple-KO cells with retroviral vectors encoding either wild-type (WT) CD4 (M3 triple-KO/CD4 cells) or the CD4_ΔCT_ molecule (M3 triple-KO/CD4_ΔCT_ cells) (Fig. 5B). The CD4_ΔCT_ molecule lacks the 25 C-terminal residues of the cytoplasmic domain, which are essential for the downregulation of CD4 by Nef (45). To formally demonstrate that the truncated CD4 on the surface of M3 triple-KO/CD4_ΔCT_ cells is resistant to Nef, the M3 triple-KO/CD4 and M3 triple-KO/CD4_ΔCT_ cells were both stably transduced with a retroviral vector encoding Nef or the empty vector. As expected, Nef clearly downregulated wild-type CD4 from the surface of M3 triple-KO/CD4 cells (Fig. 5B). In contrast, the truncated CD4 expressed by the M3 triple-KO/CD4_ΔCT_ cells was completely resistant to Nef-mediated downregulation (Fig. 5B). Notably, Nef did not enhance the specific infectivity of HIV-1_NL4-3_ virions produced in M3 triple-KO/CD4_ΔCT_ cells (Fig. 5C), as quantified using MOLT-3/ZsGreen indicator cells (57). Nevertheless, the ability of HIV-1_NL4-3_ to spread in M3 triple-KO/CD4_ΔCT_ cells after infection at a low multiplicity was highly dependent on Nef (Fig. 5D).

To determine whether the Nef-dependency of the X4-tropic HIV-1_NL4-3_ in M3 triple-KO/CD4_ΔCT_ cells is shared by R5-tropic HIV-1 viruses, we stably expressed CCR5 in these cells. The resulting M3 triple-KO/CD4_ΔCT_/CCR5 cells were infected with the R5-tropic NL-JRFL and NL-ZM109 variants of HIV-1_NL4-3_, which encode the Env proteins of primary subtype B and subtype C HIV-1 strains, respectively (26). As shown in Fig. 5E, NL-JRFL and NL-ZM109 both required Nef to spread efficiently in M3 triple-KO/CD4_ΔCT_/CCR5 cells. Taken together, these results demonstrate that Nef can profoundly enhance HIV-1 replication in the combined absence of SERINC3, SERINC5, and CD4.

To determine whether the MOLT-3 phenotype is dominant, M3 triple-KO/CD4_ΔCT_ cells were fused with JTAg double-KO cells lacking SERINC3 and SERINC5, in which Nef is not required for efficient HIV-1 replication (12). To facilitate the isolation of heterokaryons, we stably expressed one component of a split green fluorescent protein (GFP) system (GFP^1-7^) in the JTAg double-KO cells, and the other component (GFP^8-11^) in the M3 triple-KO/CD4_ΔCT_ cells. As expected, GFP expression could not be detected when JTAg double-KO(GFP^1-7^) cells were mixed at a 1:1 ratio with M3 triple-KO/CD4_ΔCT_(GFP^8-11^) cells (see Fig. S3 in the supplemental material). However, GFP expression became apparent when JTAg double-KO(GFP^1-7^) cells that had been transiently transfected with the fusogenic Newcastle disease virus (NDV) HN and F glycoproteins were mixed with M3 triple-KO/CD4_ΔCT_(GFP^8-11^) cells (Fig. S3). GFP-positive cells were isolated by fluorescent-activated cell sorting (FACS) and infected with Nef+ or Nef^−^ HIV-1_NL4-3_. As shown in Fig. 5F, virus replication was considerably more robust in the presence of Nef, consistent with the possibility that Nef counteracted an unidentified inhibitory factor.

10.1128/mbio.03382-22.3 FIG S3 Split GFP-based detection of heterokaryons. (A) JTAg double-KO(GFP_1-7_) cells (which lack SERINC3 and SERINC5) were mixed at a 1:1 ratio with M3 triple-KO/CD4_ΔCT_(GFP_8-11_) cells. (B) JTAg double-KO(GFP_1-7_) cells transiently expressing the fusogenic HN and F proteins of NDV were mixed at a 1:1 ratio with M3 triple-KO/CD4_ΔCT_(GFP_8-11_) cells. In both cases, GFP expression was examined 2 days later by flow cytometry. Download FIG S3, PDF file, 0.04 MB. Copyright © 2023 Olety et al. 2023 Olety et al. https://creativecommons.org/licenses/by/4.0/ This content is distributed under the terms of the Creative Commons Attribution 4.0 International license.

### Effect of Nef on HIV-1 replication in the triple-KO cells correlates with AP-2 binding.

HIV-1 Nef is thought to engage the AP-2 clathrin adaptor complex through conserved dileucine and diacidic motifs to accelerate the endocytosis of CD4 (9, 58–61) and to downregulate SERINC3 and SERINC5 (11, 13–15). For instance, a mutation in Nef that targets the conserved dileucine motif (LL164,165AA) abolishes binding to AP-2 and other clathrin adaptor complexes and impairs the abilities of Nef to downregulate CD4 and to counteract SERINC5 (11, 61–63). Interestingly, the LL164,165AA mutation also markedly and reproducibly reduced the ability of Nef to enhance HIV-1 replication in M3 triple-KO/CD4_ΔCT_ cells, which lack these Nef/AP-2 targets (see Fig. S4 in the supplemental material). We therefore examined the effects of additional mutations that impair the Nef/AP-2 interaction on HIV-1 replication in M3 triple-KO/CD4_ΔCT_ cells.

10.1128/mbio.03382-22.4 FIG S4 A mutation that disrupts the binding of Nef to clathrin adaptor complexes markedly impairs HIV-1 replication in M3 triple-KO/CD4_ΔCT_ cells lacking SERINCs and Nef-sensitive CD4. The cells were infected with WT (Nef^+^) HIV-1_NL4-3_ or with the LL164,165AA Nef mutant (0.2 ng p24/mL), and virus replication was examined by Western blotting of cell lysates with anti-CA (A) or by measuring p24 accumulation in the supernatants (B). The experiment was performed in duplicate (set 1 and set 2) to confirm reproducibility. Download FIG S4, PDF file, 0.2 MB. Copyright © 2023 Olety et al. 2023 Olety et al. https://creativecommons.org/licenses/by/4.0/ This content is distributed under the terms of the Creative Commons Attribution 4.0 International license.

**FIG 6:**
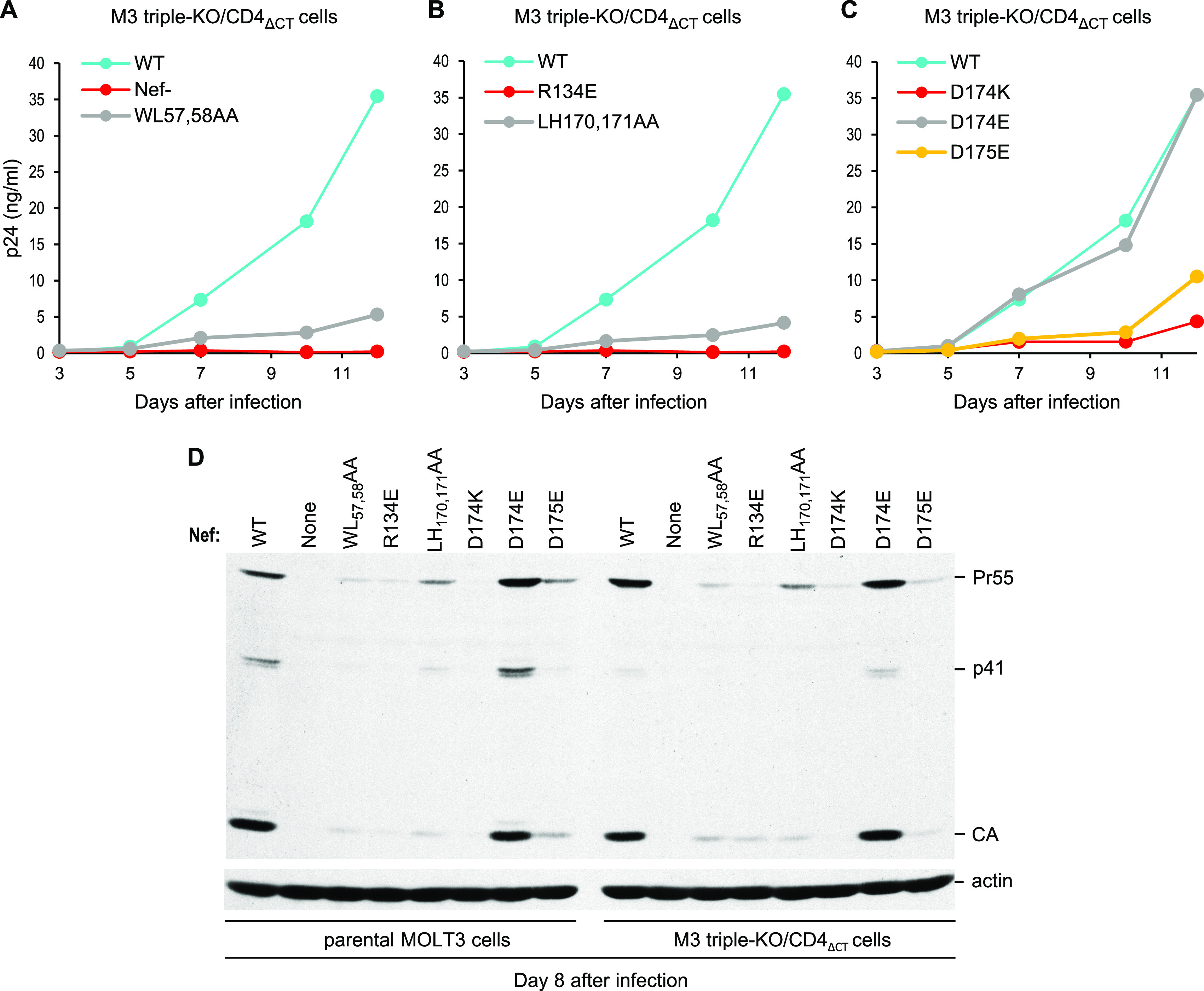
Replication of Nef mutants in M3 triple-KO/CD4_ΔCT_ cells lacking SERINCs and Nef-sensitive CD4. (A) Effect of a mutation (WL57,58AA) reported to disrupt binding of Nef to the cytoplasmic domain of CD4. (B) Effects of mutations that disrupt AP-2 binding. (C) Effects of mutations in a conserved diacidic motif that is specifically required for binding to AP-2 but not AP-1 or AP-3. Of note, AP-2 binding is unaffected by the conservative D174E substitution but is impaired by the equally conservative D175E substitution (61). (D) Side-by-side comparison of the abilities of the Nef mutants to spread in parental MOLT-3 cells and in M3 triple-KO/CD4_ΔCT_ cells. The cells were infected with WT (Nef^+^) HIV-1_NL4-3_ or with the indicated Nef mutants (0.2 ng p24/mL), and virus replication was monitored by measuring p24 accumulation in the supernatants (A to C) or by Western blotting of cell lysates with anti-CA (D). The virus replication curves are all from the same experiment.

Nef residues W57 and L58 have been implicated in the direct binding of Nef to the cytoplasmic domain of CD4 (64), and consistent with this notion, the WL57,58AA mutation disrupts the ability of Nef to downregulate CD4 while preserving the ability to downregulate MHC-I (23, 65). However, a recent crystal structure of a CD4-Nef-AP-2 complex suggests that the WL57,58AA mutation interferes with CD4 binding indirectly by affecting the positioning of the N-terminal loop of Nef at the interface between the AP-2 α subunit and the Nef core (10). In M3 triple-KO/CD4_ΔCT_ cells, the WL57,58AA mutation substantially impaired the replication of HIV-1_NL4-3_ (Fig. 6A and D; see also Fig. S5 in the supplemental material), even though the truncated CD4 expressed on these cells lacks all of the determinants involved in the interaction with Nef (10).

10.1128/mbio.03382-22.5 FIG S5 Replication of Nef mutants in MOLT-3 cells lacking SERINCs and Nef-sensitive CD4. In this repeat experiment, the abilities of Nef mutants to replicate in M3 triple-KO/CD4_ΔCT_ cells were examined as in Fig. 6D. Download FIG S5, PDF file, 0.3 MB. Copyright © 2023 Olety et al. 2023 Olety et al. https://creativecommons.org/licenses/by/4.0/ This content is distributed under the terms of the Creative Commons Attribution 4.0 International license.

A crystal structure of Nef bound to the α and σ2 subunits of AP-2 indicates that a salt bridge between R134 and D175 stabilizes the conformation of the Nef C-terminal loop, which is directly involved in AP-2 binding (66). Consistent with this model, an R134E mutation eliminated the ability of Nef to bind AP-2 and to downregulate CD4 (66). Interestingly, the R134E mutation also abrogated the ability of Nef to enhance HIV-1 replication in M3 triple-KO/CD4_ΔCT_ cells (Fig. 6B and D; see also Fig. S5).

In addition to the critical dileucine and diacidic motifs, the C-terminal loop of Nef contains other determinants that are involved in the association with AP-2 (66, 67). In particular, mutations in a hydrophobic region downstream of the dileucine motif impaired both AP-2 binding and CD4 downregulation (67). For instance, the LH170,171AA mutation within this region impaired AP-2 binding and abrogated CD4 downregulation by Nef (67). The LH170,171AA mutation also clearly impaired the replication of HIV-1_NL4-3_ in M3 triple-KO/CD4_ΔCT_ cells (Fig. 6B and D; see also Fig. S5).

We previously reported that a point mutation (D174K) in the conserved diacidic motif of Nef substantially impaired its ability to enhance HIV-1 replication in wild-type MOLT-3 cells (45) and now find that the D174K mutation has a comparable inhibitory effect on HIV-1 replication in M3 triple-KO/CD4_ΔCT_ cells (Fig. 6C and D; see also Fig. S5). In contrast, HIV-1_NL4-3_ with a conservative substitution at the same position of Nef (D174E) replicated with wild-type kinetics in M3 triple-KO/CD4_ΔCT_ cells (Fig. 6C and D; see also Fig. S5). Of note, the D174E mutation also did not affect the binding of Nef to clathrin adaptor complexes (61). In contrast, a conservative substitution at position 175 of Nef (D175E) reduced binding to AP-2 without affecting binding to AP-1 or AP-3 (61). Interestingly, the D175E mutation clearly delayed HIV-1 replication in M3 triple-KO/CD4_ΔCT_ cells (Fig. 6C and D; see also Fig. S5). Thus, the effects of point mutations in the diacidic motif on HIV-1 replication correlated with their effects on AP-2 binding.

To examine whether the presence of SERINCs and of Nef-sensitive CD4 affected the phenotypes of these Nef mutants, we performed a side-by-side comparison of their abilities to spread in parental MOLT-3 cells and in M3 triple-KO/CD4_ΔCT_ cells. As shown in Fig. 6D, each mutant behaved similarly in both cellular contexts, consistent with the proposal that the requirement for Nef in MOLT-3 cells is primarily determined by an unknown restriction factor (45).

### Effect of Nef on HIV-1 replication in the triple-KO cells depends on AP-2.

**FIG 7:**
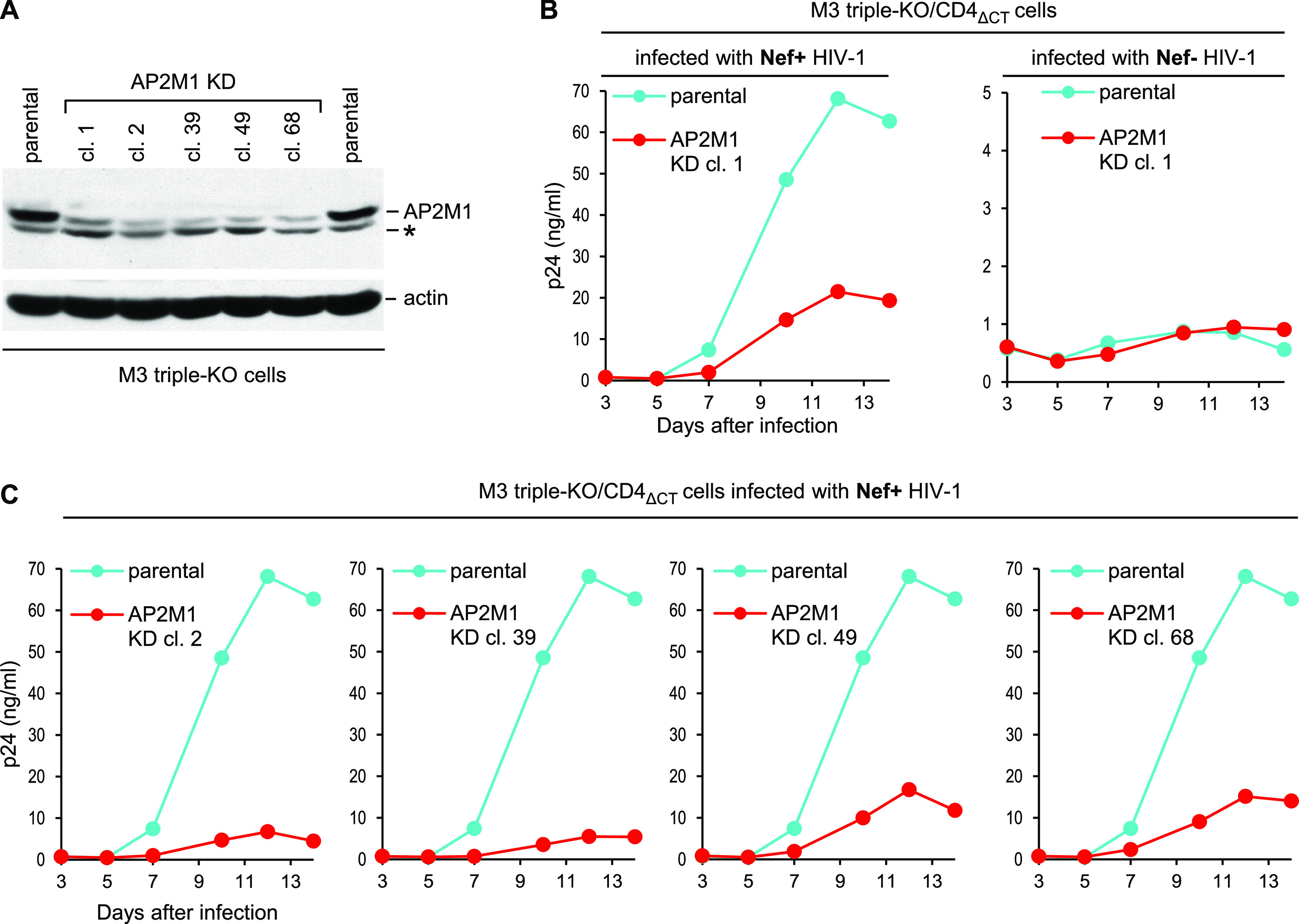
Knockdown of AP-2 subunit AP2M1 impairs WT HIV-1 replication in cells lacking SERINCs and Nef-sensitive CD4. (A) AP2M1 expression in parental M3 triple-KO (SERINC3/SERINC5/CD4 KO) cells and in M3 triple-KO-derived AP2M1 knockdown (KD) clones analyzed by Western blotting. The asterisk indicates a nonspecific band. (B and C) Replication of Nef^+^ (B and C) and Nef^−^ (B) HIV-1_NL4-3_ in parental M3 triple-KO cells and in M3 triple-KO-derived AP2M1 KD clones expressing the Nef-resistant CD4_ΔCT_ molecule. The cells were infected with 0.2 ng p24/mL, and virus replication was monitored by measuring p24 accumulation in the supernatants.

The results described above specifically implicated AP-2 in the ability of Nef to enhance HIV-1 replication in MOLT-3 cells lacking the Nef/AP-2 targets SERINC3, SERINC5, and CD4. To confirm the role of AP-2, we subjected M3 triple-KO cells to a further round of CRISPR/Cas9-mediated gene editing with an sgRNA targeting AP2M1, the μ2 subunit of AP-2. Although this approach did not yield any cells that completely lacked AP2M1, we obtained several clones that expressed substantially reduced amounts (Fig. 7A). Of note, cells that completely lacked AP2M1 also could not be obtained in a previous study that used the same sgRNA, probably because a small amount of AP-2 is essential for viability (68).

To allow HIV-1 entry, the M3 triple-KO cell-derived AP2M1 knockdown (KD) clones, which lack endogenous CD4, were reconstituted with the Nef-resistant CD4_ΔCT_ molecule. As shown in Fig. 7B, WT (Nef^+^) HIV-1_NL4-3_ spread less efficiently in the AP2M1 KD clone 1 than in the parental cells, whereas Nef-deficient HIV-1_NL4-3_ failed to spread even in the parental cells. To confirm that the enhancement of HIV-1 spreading by Nef depended on AP-2, we monitored the replication of WT (Nef^+^) HIV-1_NL4-3_ in the remaining four AP2M1 KD clones and observed that it was impaired in each case (Fig. 7C).

**FIG 8:**
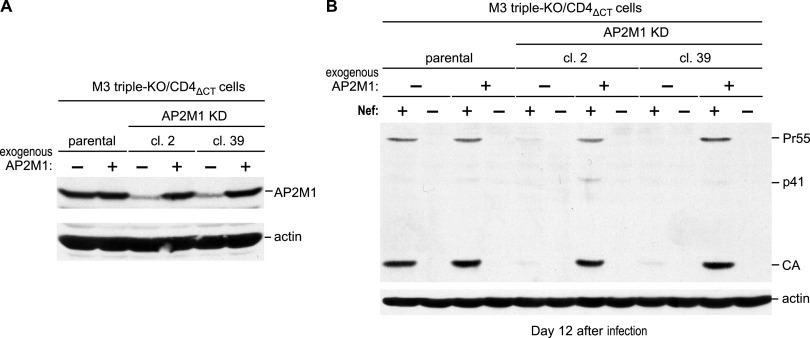
Restoration of AP2M1 expression rescues WT HIV-1 replication in cells lacking SERINCs and Nef-sensitive CD4. (A) Western blots showing AP2M1 expression in parental M3 triple-KO/CD4_ΔCT_ cells and in M3 triple-KO-derived AP2M1 KD/CD4_ΔCT_ clones stably transduced with the empty pCX4pur retroviral vector or with a version expressing AP2M1. (B) Replication of WT (Nef^+^) and Nef^−^ HIV-1_NL4-3_ in the same cells. The cells were infected with 0.2 ng p24/mL, and cell lysates were examined with anti-CA and anti-actin 12 days after infection.

To exclude off-target effects, the parental M3 triple-KO/CD4_ΔCT_ cells and two KD clones with relatively low residual AP2M1 expression levels (clones 2 and 39) were stably transduced with a retroviral vector encoding AP2M1. Western blotting showed that while AP2M1 levels in the parental cells were only minimally affected, AP2M1 expression in both KD clones was fully restored (Fig. 8A). Importantly, while the ectopic expression of AP2M1 in the parental cells had no discernible effect on HIV-1 replication, it restored WT (Nef^+^) HIV-1_NL4-3_ replication in both AP2M1 KD clones to levels comparable to those in the parental cells (Fig. 8B).

Because Nef-deficient HIV-1_NL4-3_ did not replicate to detectable levels in any of the cultures analyzed (Fig. 8B), we conducted another experiment in which infections with Nef-deficient HIV-1_NL4-3_ were started with a 10-fold higher amount of input virus. Even under these conditions, only a slight increase in extracellular p24 antigen concentrations over a period of 3 weeks could be detected after infection of AP2M1 clone 2 cells (see Fig. S6 in the supplemental material). While virus production also remained modest after infection of AP2M1 clone 39 cells, a clear rise in extracellular p24 levels nevertheless became evident after an initial lag period (Fig. S6). Notably, extracellular p24 levels after infection with Nef-deficient HIV-1_NL4-3_ were comparable in the presence and absence of ectopically expressed AP2M1 (Fig. S6). Taken together, these results confirm that AP-2 is crucial for the efficient replication of Nef^+^ HIV-1 in MOLT-3 cells that lack the Nef/AP-2 targets SERINC3, SERINC5, and CD4.

10.1128/mbio.03382-22.6 FIG S6 Replication of Nef-deficient HIV-1 in M3 triple-KO-derived AP2M1 KD/CD4_ΔCT_ clones stably transduced with the empty pCX4pur retroviral vector or with a version expressing AP2M1. The cells were infected with a relatively high amount of input virus (2 ng p24/mL), and virus replication was examined by measuring p24 accumulation in the supernatants. Note the log scale of the *y* axes. Download FIG S6, PDF file, 0.1 MB. Copyright © 2023 Olety et al. 2023 Olety et al. https://creativecommons.org/licenses/by/4.0/ This content is distributed under the terms of the Creative Commons Attribution 4.0 International license.

## DISCUSSION

HIV-1 and simian immunodeficiency virus Nef proteins enhance virus replication in primary CD4^+^ T cells, particularly if these are infected before stimulation, but the molecular basis for this biological activity is incompletely understood. In Jurkat E6.1 T lymphoid cells, Nef appears to enhance HIV-1 replication primarily by counteracting SERINC5 because the unrelated SERINC antagonists Nef and glycoGag supported HIV-1 replication in these cells to a similar extent (45). Consistent with this observation, Nef is critical for HIV-1 propagation in Jurkat-derived JTAg cells but not in double-KO JTAg cells lacking SERINC3 and SERINC5 (12). However, the potent SERINC5 antagonist glycoGag was unable to substitute for Nef in supporting HIV-1 replication in MOLT-3 T lymphoid cells (45). Furthermore, HIV-1 replication remained highly dependent on Nef in MOLT-3 cells lacking SERINC3 and SERINC5 (45). Together, these observations suggested that MOLT-3 cells express another factor that is affected by Nef but not glycoGag. Since MOLT-3 cells resemble primary human PBMC in this regard (45), we have now used MOLT-3 cells as a model system to explore the possible involvement of other host factors through CRISPR/Cas9-mediated gene editing.

One highly conserved activity of lentiviral Nef proteins is the relocalization of the T cell receptor-proximal kinase LCK to an intracellular compartment (69). Nef inhibits the targeting of LCK to the immunological synapse, possibly to limit T cell signaling to prevent activation-induced cell death (36). Instead, the Nef-induced retargeting of LCK triggers intracellular signaling to facilitate HIV-1 replication in primary human T lymphocytes (37). Furthermore, the association of Nef and LCK was found to be important for the enhancement of HIV-1 replication by Nef in a T cell line (46). In contrast, we now find that LCK is entirely dispensable for the stimulation of HIV-1 replication by Nef in MOLT-3 cells. Since HIV-1 replication in these cells is highly dependent on Nef (45), we conclude that Nef can profoundly enhance HIV-1 spreading in an LCK-independent manner.

The ability of Nef to interact with PAK2 is generally conserved among the different HIV-1 groups, indicating that the interaction is of biological significance (41). It has been reported that Nef-associated PAK induces antiapoptotic signaling in T cells and, thus, supports virus replication by decreasing HIV-1-induced cell death (52). Furthermore, it has been proposed that Nef enhances virus replication by engaging PAK2 to enhance the responsiveness of infected T cells to activating stimuli (43). In addition, Nef reduces the motility of infected T cells in a PAK2-dependent manner (42, 70, 71), possibly to facilitate the formation of stable virological synapses and thus the cell-to-cell transmission of HIV-1 (54). PAK2 also appears essential for the association of Nef with components of the exocyst complex (72), which has been suggested to contribute to the intercellular spread of HIV-1 via tunneling nanotubes (73). However, it is evident from the results of the present study that Nef can substantially enhance HIV-1 spreading in some other way because we find that the effect of Nef on HIV-1 spreading in MOLT-3 cells does not depend on PAK2 or any other group I PAK.

Consistent with the observation that CD4 on the surface of cells infected with HIV-1 can reduce the infectivity of progeny virions (21), it has been noted that the ability of Nef to enhance HIV-1 replication in primary T cells or in *ex vivo* lymphoid tissue correlates with its ability to downregulate CD4 (22, 74). Furthermore, among several Nef activities, the ability to downregulate CD4 correlated most closely with the enhancement of HIV-1 pathogenicity in humanized mouse models (23, 24). In agreement with these reports, we observed previously that mutations that impaired the ability of Nef to downregulate CD4 also impaired its ability to promote HIV-1 replication in MOLT-3 cells (45). Conversely, we observed that Nef remained fully capable of enhancing HIV-1 replication in MOLT-3 cells overexpressing a Nef-resistant CD4 molecule (45). However, since these cells also expressed Nef-sensitive CD4 endogenously, we could not strictly rule out that the downregulation of the endogenous CD4 accounted for the effect of Nef on HIV-1 spreading (45). In the present study, we observed that Nef substantially enhanced HIV-1 replication even in triple-KO MOLT-3 cells that simultaneously lacked the three Nef targets SERINC3, SERINC5, and CD4 and expressed exclusively a Nef-resistant CD4 to allow HIV-1 entry. We conclude that the downregulation of CD4 is dispensable for the pronounced effect of Nef on HIV-1 spreading in MOLT-3 cells. Furthermore, SERINCs and CD4 do not act in a redundant manner to restrict the propagation of Nef-deficient HIV-1 in these cells.

Nef engages the AP-2 adaptor complex to downregulate both CD4 and SERINCs (9, 11, 13, 14, 58–60) and the AP-1 adaptor complex to decrease MHC-I surface levels (19, 75, 76). Notably, HIV-1 replication in MOLT-3 cells is markedly impaired by the LL164,164AA mutation in a conserved dileucine motif of Nef that disrupts binding to AP-2 and other clathrin adaptor complexes (45, 77, 78) and abrogates the ability of Nef to downregulate both CD4 and SERINC5 (11, 62). Similarly, HIV-1 replication in MOLT-3 cells is impaired by the DD174,175AA mutation in a conserved diacidic motif of Nef that specifically prevents Nef from binding to AP-2 but not AP-1 hemicomplexes and disrupts its ability to downregulate CD4 (23, 45, 77). Together, these observations implicated AP-2 in the enhancement of HIV-1 replication in MOLT-3 cells. We now find that various point mutations in Nef that have been reported to compromise its ability to interact with AP-2 impair HIV-1 replication even in MOLT-3 cells that lack the three Nef/AP-2 targets SERINC3, SERINC5, and CD4. Instead of endogenous CD4, these cells expressed a CD4 that lacks cytoplasmic tail sequences required for the downregulation of CD4 by Nef (Fig. 5) but not for its ability to support HIV-1 entry (79). In particular, the phenotypes resulting from conservative substitutions within the D174/175 diacidic motif of Nef appear noteworthy. On the one hand, the D174E mutation, which does not affect AP-2 binding (61), had no effect on the ability of Nef to enhance HIV-1 replication. On the other hand, the equally conservative D175E substitution, which selectively impairs binding to AP-2 but not AP-1 or AP-3 (61), compromised HIV-1 replication both in the presence or absence of SERINC3, SERINC5, and Nef-sensitive CD4. Thus, the effects of mutations in the diacidic motif on HIV-1 replication correlated remarkably well with their effects on AP-2 binding.

CRISPR/Cas9-mediated gene editing of the AP2M1 subunit of AP-2 has been used previously to study protein trafficking in HeLa cells (68). Although a complete knockout could not be achieved, presumably because AP-2 is essential for cell viability, the approach yielded cells with substantially reduced AP2M1 expression and marked defects in transferrin receptor endocytosis (68). Based on these observations, we used the CISPR/Cas9 approach to target AP2M1 in MOLT-3 cells that already lacked SERINC3, SERINC5, and Nef-sensitive CD4 and were able to obtain several clones with clearly reduced AP2M1 expression levels. Interestingly, the ability of Nef^+^ HIV-1 to replicate was compromised in all clones. The spreading of Nef^+^ HIV-1 could be fully rescued through the ectopic expression of AP2M1, confirming that AP-2 plays an important role in this model system.

Taken together, our observations imply that the potent effect of Nef on HIV-1 replication in MOLT-3 cells depends on AP-2 even in the absence of several known Nef targets that have the potential to restrict HIV-1 and are downregulated in an AP-2-dependent manner. They thus suggest that Nef additionally engages AP-2 to control the trafficking of an unknown factor that affects HIV-1 spreading.

## MATERIALS AND METHODS

### HIV-1 proviral constructs.

NL4-3/Nefstop is a *nef*-deficient variant of the full-length, X4-tropic HIV-1 molecular clone pNL4-3 (GenBank accession number M19921) that has *nef* codons 31 to 33 replaced by three consecutive premature termination codons (12). The full-length HIV-1 proviral constructs NL-JRFL and NL-ZM109 (both Nef^+^) are R5-tropic variants of pNL4-3 that have been described (26, 45). Full-length variants of pNL4-3 with point mutations in *nef* were obtained by inserting mutant *nef* sequences generated by a PCR-based approach between unique XhoI and NcoI sites of pNL4-3.

### Retroviral vectors.

The retroviral vectors pCXbsrCD4 and pCXbsrCD4_ΔCT_ encode full-length human CD4 and a version that lacks most of the cytoplasmic domain, respectively (45). To obtain a retroviral vector expressing HIV-1 Nef, the *nef* gene of HIV-1_LAI_ (GenBank accession number K02013) was inserted between the unique BglII and EcoRI sites of MSCVpuro (Clontech). The retroviral vector pCX4pur-synCCR5 encodes codon-optimized human CCR5 (45). The coding sequence for human AP2M1 (GenBank accession number BC004996) was cloned into the retroviral vector pCX4pur (80). The retroviral vector pCX4pur-Rluc8-GFP^1-7^ encodes Renilla luciferase variant 8 (Rluc8) residues 1 to 155 fused to GFP residues 2 to 156. The retroviral vector pCX4pur-Rluc8-GFP^8-11^ encodes GFP residues 157 to 230 fused to Rluc8 residues 156 to 311. The Rluc8-GFP coding sequences were amplified from phRL-CMV-based plasmids (81) and cloned into pCX4pur.

### KO cells.

MOLT-3 cells were obtained from the ATCC. To obtain MOLT-3 cells lacking LCK, preassembled ribonucleotide complexes consisting of purified Cas9 (Synthego) and chemically modified synthetic sgRNA (target sequence: 5′-GCTCCGCGTCCTTGCGGCTC-3′) (Synthego) were delivered into MOLT-3 cells via nucleofection using the Cell Line Nucleofector kit V with the Nucleofector II device (Lonza). Clones obtained by limiting dilution were screened by Western blotting with anti-LCK antibody 3A5 (Santa Cruz Biotechnology; sc-433). Protein loading was assessed with anti-actin antibody (Santa Cruz Biotechnology; sc-47778).

MOLT-3 cells lacking PAK2 were obtained by transiently transfecting a plasmid expressing an sgRNA targeting PAK2 (target sequence, 5′-GATTTCGTATGATCCGGTCG-3′) by nucleofection, along with a plasmid expressing Cas9. Clones obtained by limiting dilution were screened by Western blotting with anti-PAK2 (Cell Signaling Technology; 2608) and anti-actin antibodies. To obtain MOLT-3 cells lacking all group I PAKs, Cas9/sgRNA complexes targeting PAK1 (target sequence, 5′-AGGCACCGTGTACACAGCAA-3′) were delivered into PAK2 KO MOLT-3 cells by nucleofection. Clones obtained by limiting dilution were screened by Western blotting with anti-PAK1 (Cell Signaling Technology; 2602) and anti-actin antibodies.

MOLT-3 double-KO cells lacking SERINC3 and SERINC5 (MOLT-3 S3/5 double-KO cells) have been described (26). To obtain MOLT-3 cells lacking SERINC3, SERINC5, and CD4 (M3 triple-KO cells), Cas9/sgRNA complexes targeting CD4 (target sequence, 5′-GAGGTGCAATTGCTAGTGTT-3′) were delivered into MOLT-3 S3/5 double-KO cells by nucleofection. Clones obtained by limiting dilution were screened by flow cytometry after staining with anti-CD4 antibody (BioLegend; 300502) and PE-conjugated secondary antibody (Jackson ImmunoResearch; 115-116-146).

M3 triple-KO/CD4 and M3 triple-KO/CD4_ΔCT_ cells were obtained by retroviral transduction of M3 triple-KO cells with pCXbsrCD4 and pCXbsrCD4_ΔCT_, respectively, followed by selection with blasticidin (5 μg/mL). The ectopic expression of CD4 or CD4_ΔCT_ was confirmed by flow cytometry. To examine the effects of Nef on CD4 surface levels, M3 triple-KO/CD4 and M3 triple-KO/CD4_ΔCT_ cells were transduced with empty MSCVpuro or MSCVpuroNef_LAI_, followed by selection with puromycin (1 μg/mL). To facilitate the entry of R5-tropic viruses, M3 triple-KO/CD4_ΔCT_ cells were transduced with pCX4pur-synCCR5, followed by selection with puromycin (1 μg/mL).

M3 triple-KO cells expressing reduced amounts of the μ2 subunit of AP-2 (AP2M1) were obtained by delivering Cas9/sgRNA complexes targeting AP2M1 (target sequence, 5′-CGATGTCATCTCGGTAGACT-3′) into M3 triple-KO cells by nucleofection. Clones obtained by limiting dilution were screened by Western blotting with anti-AP50 (BD Biosciences; 611351) and anti-actin antibodies. To restore AP2M1 expression, clones expressing reduced amounts of AP2M1 were transduced with pCX4purAP2M1, followed by selection with puromycin (1 μg/mL).

### Cell fusion.

JTAg double-KO cells, which lack SERINC3 and SERINC5 and express only low levels of CD4 (12), were transduced with pCX4pur-Rluc8-GFP^1-7^, and M3 triple-KO/CD4_ΔCT_ cells were transduced with pCX4pur-Rluc8-GFP^8-11^, followed by selection with puromycin (1 μg/mL). To transiently express the NDV hemagglutinin-neuraminidase (HN) and fusion (F) proteins, JTAg double-KO cells expressing Rluc8-GFP^1-7^ were cotransfected with pCAGGS-HN and pCAGGS-F using Lipofectamine 2000 (Invitrogen). The transfected cells were then mixed with M3 triple-KO/CD4_ΔCT_ cells expressing Rluc8-GFP^8-11^. After 2 days of incubation, GFP-positive cells were sorted with a Sony MA900 cell sorter.

### Virus replication studies.

Replication-competent HIV-1 was produced by transfecting 293T cells with plasmids containing full-length proviruses. Virus containing supernatants were clarified by low-speed centrifugation, passed through 0.45-μm filters, normalized for HIV-1 capsid (p24) antigen with an HIV-1 p24 enzyme-linked immunosorbent assay (ELISA) kit (XpressBio), and used to infect target cells.

MOLT-3 cells were infected in T25 flasks in 5 mL medium. FACS-sorted cells were infected in 96-well plates in 200 μL medium. PBMC were obtained from LRS chambers by Ficoll-Hypaque density gradient centrifugation and kept in RPMI 1640 medium supplemented with 15% human AB serum (Millipore Sigma). CD4^+^ T cells were purified from fresh PBMC by negative selection with an EasySep human CD4^+^ T cell enrichment kit (Stemcell Technologies). PBMC were seeded into 12-well plates in 2 mL medium and immediately stimulated with 2 μg/mL phytohemagglutinin (PHA) (Millipore Sigma). One day later, the PHA-containing medium was replaced by fresh medium, and the cells were infected in the presence of 20 U/mL interleukin 2 (Roche). CD4^+^ T cells were seeded into 12-well plates and immediately infected at a p24 concentration of 1 ng/mL. On day 3 after infection, the cells were stimulated with 2 μg/mL PHA. The next day, the PHA-containing medium was replaced by fresh medium containing 20 U/mL interleukin 2.

Virus replication was monitored by comparing Gag protein expression levels in infected cells by Western blotting using anti-HIV-1 capsid (CA) antibody 183-H12-5C and anti-actin and/or by measuring the accumulation of p24 antigen in the culture supernatants over time with an HIV-1 p24 ELISA kit (XpressBio).

### Analysis of virus infectivity.

Vesicular stomatitis virus G (VSV-G) pseudotypes of NL4-3 or NL4-3/Nefstop were obtained by transfecting 293T cells, clarified by low-speed centrifugation, passed through 0.45-μm filters, normalized for p24 antigen, and used to infect M3 triple-KO/CD4_ΔCT_ cells. After overnight incubation, the infected M3 triple-KO/CD4_ΔCT_ cells were extensively washed to remove input virus, and resuspended in fresh medium. Supernatants harvested 24 h later were clarified by low-speed centrifugation, passed through 0.45-μm filters, normalized for p24 antigen, and used to infect MOLT-3/ZsGreen indicator cells (clone 45), which turn bright green upon infection (57). To limit virus replication to a single cycle, the entry inhibitor ADM3100 (5 μM) was added 16 h later. After another 2 days, the cells were fixed with 4% paraformaldehyde, and ZsGreen expression was analyzed by flow cytometry.

## References

[1] Kestler HW, III, Ringler DJ, Mori K, Panicali DL, Sehgal PK, Daniel MD, Desrosiers RC. 1991. Importance of the nef gene for maintenance of high virus loads and for development of AIDS. Cell 65:651–662. doi:10.1016/0092-8674(91)90097-i. 2032289

[2] Deacon NJ, Tsykin A, Solomon A, Smith K, Ludford-Menting M, Hooker DJ, McPhee DA, Greenway AL, Ellett A, Chatfield C, Lawson VA, Crowe S, Maerz A, Sonza S, Learmont J, Sullivan JS, Cunningham A, Dwyer D, Dowton D, Mills J. 1995. Genomic structure of an attenuated quasi species of HIV-1 from a blood transfusion donor and recipients. Science 270:988–991. doi:10.1126/science.270.5238.988. 7481804

[3] Miller MD, Warmerdam MT, Gaston I, Greene WC, Feinberg MB. 1994. The human immunodeficiency virus-1 nef gene product: a positive factor for viral infection and replication in primary lymphocytes and macrophages. J Exp Med 179:101–113. doi:10.1084/jem.179.1.101. 8270859 PMC2191317

[4] Spina CA, Kwoh TJ, Chowers MY, Guatelli JC, Richman DD. 1994. The importance of nef in the induction of human immunodeficiency virus type 1 replication from primary quiescent CD4 lymphocytes. J Exp Med 179:115–123. doi:10.1084/jem.179.1.115. 7903679 PMC2191324

[5] Munch J, Rajan D, Schindler M, Specht A, Rucker E, Novembre FJ, Nerrienet E, Muller-Trutwin MC, Peeters M, Hahn BH, Kirchhoff F. 2007. Nef-mediated enhancement of virion infectivity and stimulation of viral replication are fundamental properties of primate lentiviruses. J Virol 81:13852–13864. doi:10.1128/JVI.00904-07. 17928336 PMC2168858

[6] Garcia JV, Miller AD. 1991. Serine phosphorylation-independent downregulation of cell-surface CD4 by nef. Nature 350:508–511. doi:10.1038/350508a0. 2014052

[7] Mariani R, Skowronski J. 1993. CD4 down-regulation by nef alleles isolated from human immunodeficiency virus type 1-infected individuals. Proc Natl Acad Sci USA 90:5549–5553. doi:10.1073/pnas.90.12.5549. 8516299 PMC46758

[8] Aiken C, Konner J, Landau NR, Lenburg ME, Trono D. 1994. Nef induces CD4 endocytosis: requirement for a critical dileucine motif in the membrane-proximal CD4 cytoplasmic domain. Cell 76:853–864. doi:10.1016/0092-8674(94)90360-3. 8124721

[9] Chaudhuri R, Lindwasser OW, Smith WJ, Hurley JH, Bonifacino JS. 2007. Downregulation of CD4 by human immunodeficiency virus type 1 Nef is dependent on clathrin and involves direct interaction of Nef with the AP2 clathrin adaptor. J Virol 81:3877–3890. doi:10.1128/JVI.02725-06. 17267500 PMC1866153

[10] Kwon Y, Kaake RM, Echeverria I, Suarez M, Karimian Shamsabadi M, Stoneham C, Ramirez PW, Kress J, Singh R, Sali A, Krogan N, Guatelli J, Jia X. 2020. Structural basis of CD4 downregulation by HIV-1 Nef. Nat Struct Mol Biol 27:822–828. doi:10.1038/s41594-020-0463-z. 32719457 PMC7483821

[11] Rosa A, Chande A, Ziglio S, De Sanctis V, Bertorelli R, Goh SL, McCauley SM, Nowosielska A, Antonarakis SE, Luban J, Santoni FA, Pizzato M. 2015. HIV-1 Nef promotes infection by excluding SERINC5 from virion incorporation. Nature 526:212–217. doi:10.1038/nature15399. 26416734 PMC4861059

[12] Usami Y, Wu Y, Gottlinger HG. 2015. SERINC3 and SERINC5 restrict HIV-1 infectivity and are counteracted by Nef. Nature 526:218–223. doi:10.1038/nature15400. 26416733 PMC4600458

[13] Shi J, Xiong R, Zhou T, Su P, Zhang X, Qiu X, Li H, Li S, Yu C, Wang B, Ding C, Smithgall TE, Zheng YH. 2018. HIV-1 Nef antagonizes SERINC5 restriction by downregulation of SERINC5 via the endosome/lysosome system. J Virol 92:e00196-18. doi:10.1128/JVI.00196-18. 29514909 PMC5952139

[14] Staudt RP, Smithgall TE. 2020. Nef homodimers down-regulate SERINC5 by AP-2-mediated endocytosis to promote HIV-1 infectivity. J Biol Chem 295:15540–15552. doi:10.1074/jbc.RA120.014668. 32873704 PMC7667984

[15] Buffalo CZ, Iwamoto Y, Hurley JH, Ren X. 2019. How HIV Nef proteins hijack membrane traffic to promote infection. J Virol 93:e01322-19. doi:10.1128/JVI.01322-19. 31578291 PMC6880166

[16] Schwartz O, Marechal V, Le Gall S, Lemonnier F, Heard JM. 1996. Endocytosis of major histocompatibility complex class I molecules is induced by the HIV-1 Nef protein. Nat Med 2:338–342. doi:10.1038/nm0396-338. 8612235

[17] Veillette M, Desormeaux A, Medjahed H, Gharsallah NE, Coutu M, Baalwa J, Guan Y, Lewis G, Ferrari G, Hahn BH, Haynes BF, Robinson JE, Kaufmann DE, Bonsignori M, Sodroski J, Finzi A. 2014. Interaction with cellular CD4 exposes HIV-1 envelope epitopes targeted by antibody-dependent cell-mediated cytotoxicity. J Virol 88:2633–2644. doi:10.1128/JVI.03230-13. 24352444 PMC3958102

[18] Collins KL, Chen BK, Kalams SA, Walker BD, Baltimore D. 1998. HIV-1 Nef protein protects infected primary cells against killing by cytotoxic T lymphocytes. Nature 391:397–401. doi:10.1038/34929. 9450757

[19] Jia X, Singh R, Homann S, Yang H, Guatelli J, Xiong Y. 2012. Structural basis of evasion of cellular adaptive immunity by HIV-1 Nef. Nat Struct Mol Biol 19:701–706. doi:10.1038/nsmb.2328. 22705789 PMC3407041

[20] Ross TM, Oran AE, Cullen BR. 1999. Inhibition of HIV-1 progeny virion release by cell-surface CD4 is relieved by expression of the viral Nef protein. Curr Biol 9:613–621. doi:10.1016/s0960-9822(99)80283-8. 10375525

[21] Lama J, Mangasarian A, Trono D. 1999. Cell-surface expression of CD4 reduces HIV-1 infectivity by blocking Env incorporation in a Nef- and Vpu-inhibitable manner. Curr Biol 9:622–631. doi:10.1016/S0960-9822(99)80284-X. 10375528

[22] Glushakova S, Munch J, Carl S, Greenough TC, Sullivan JL, Margolis L, Kirchhoff F. 2001. CD4 down-modulation by human immunodeficiency virus type 1 Nef correlates with the efficiency of viral replication and with CD4(+) T-cell depletion in human lymphoid tissue ex vivo. J Virol 75:10113–10117. doi:10.1128/JVI.75.21.10113-10117.2001. 11581379 PMC114585

[23] Stoddart CA, Geleziunas R, Ferrell S, Linquist-Stepps V, Moreno ME, Bare C, Xu W, Yonemoto W, Bresnahan PA, McCune JM, Greene WC. 2003. Human immunodeficiency virus type 1 Nef-mediated downregulation of CD4 correlates with Nef enhancement of viral pathogenesis. J Virol 77:2124–2133. doi:10.1128/jvi.77.3.2124-2133.2003. 12525647 PMC140869

[24] Watkins RL, Zou W, Denton PW, Krisko JF, Foster JL, Garcia JV. 2013. In vivo analysis of highly conserved Nef activities in HIV-1 replication and pathogenesis. Retrovirology 10:125. doi:10.1186/1742-4690-10-125. 24172637 PMC3907037

[25] Matheson NJ, Sumner J, Wals K, Rapiteanu R, Weekes MP, Vigan R, Weinelt J, Schindler M, Antrobus R, Costa AS, Frezza C, Clish CB, Neil SJ, Lehner PJ. 2015. Cell surface proteomic map of HIV infection reveals antagonism of amino acid metabolism by Vpu and Nef. Cell Host Microbe 18:409–423. doi:10.1016/j.chom.2015.09.003. 26439863 PMC4608997

[26] Olety B, Peters P, Wu Y, Usami Y, Gottlinger H. 2021. HIV-1 propagation is highly dependent on basal levels of the restriction factor BST2. Sci Adv 7:eabj7398. doi:10.1126/sciadv.abj7398. 34714669 PMC8555903

[27] Zeng C, Waheed AA, Li T, Yu J, Zheng YM, Yount JS, Wen H, Freed EO, Liu SL. 2021. SERINC proteins potentiate antiviral type I IFN production and proinflammatory signaling pathways. Sci Signal 14:eabc7611. doi:10.1126/scisignal.abc7611. 34520227 PMC9549701

[28] Janaka SK, Palumbo AV, Tavakoli-Tameh A, Evans DT. 2021. Selective disruption of SERINC5 antagonism by Nef impairs SIV replication in primary CD4(+) T cells. J Virol 95:e01911-20. doi:10.1128/JVI.01911-20. 33504599 PMC8103682

[29] Markle TJ, Philip M, Brockman MA. 2013. HIV-1 Nef and T-cell activation: a history of contradictions. Future Virol 8:391–404. doi:10.2217/fvl.13.20. PMC3810967 24187576

[30] Saksela K, Cheng G, Baltimore D. 1995. Proline-rich (PxxP) motifs in HIV-1 Nef bind to SH3 domains of a subset of Src kinases and are required for the enhanced growth of Nef+ viruses but not for down-regulation of CD4. EMBO J 14:484–491. doi:10.1002/j.1460-2075.1995.tb07024.x. 7859737 PMC398106

[31] Staudt RP, Alvarado JJ, Emert-Sedlak LA, Shi H, Shu ST, Wales TE, Engen JR, Smithgall TE. 2020. Structure, function, and inhibitor targeting of HIV-1 Nef-effector kinase complexes. J Biol Chem 295:15158–15171. doi:10.1074/jbc.REV120.012317. 32862141 PMC7606690

[32] Emert-Sedlak LA, Moukha-Chafiq O, Shi H, Du S, Alvarado JJ, Pathak V, Tanner SG, Hunter RN, Nebane M, Chen L, Ilina TV, Ishima R, Zhang S, Kuzmichev YV, Wonderlich ER, Schader SM, Augelli-Szafran CE, Ptak RG, Smithgall TE. 2022. Inhibitors of HIV-1 Nef-mediated activation of the myeloid Src-family kinase Hck block HIV-1 replication in macrophages and disrupt MHC-I downregulation. ACS Infect Dis 8:91–105. doi:10.1021/acsinfecdis.1c00288. 34985256 PMC9274903

[33] Collette Y, Dutartre H, Benziane A, Ramos M, Benarous R, Harris M, Olive D. 1996. Physical and functional interaction of Nef with Lck. HIV-1 Nef-induced T-cell signaling defects. J Biol Chem 271:6333–6341. doi:10.1074/jbc.271.11.6333. 8626429

[34] Greenway A, Azad A, Mills J, McPhee D. 1996. Human immunodeficiency virus type 1 Nef binds directly to Lck and mitogen-activated protein kinase, inhibiting kinase activity. J Virol 70:6701–6708. doi:10.1128/JVI.70.10.6701-6708.1996. 8794306 PMC190712

[35] Baur AS, Sass G, Laffert B, Willbold D, Cheng-Mayer C, Peterlin BM. 1997. The N-terminus of Nef from HIV-1/SIV associates with a protein complex containing Lck and a serine kinase. Immunity 6:283–291. doi:10.1016/S1074-7613(00)80331-3. 9075929

[36] Thoulouze MI, Sol-Foulon N, Blanchet F, Dautry-Varsat A, Schwartz O, Alcover A. 2006. Human immunodeficiency virus type-1 infection impairs the formation of the immunological synapse. Immunity 24:547–561. doi:10.1016/j.immuni.2006.02.016. 16713973

[37] Pan X, Rudolph JM, Abraham L, Habermann A, Haller C, Krijnse-Locker J, Fackler OT. 2012. HIV-1 Nef compensates for disorganization of the immunological synapse by inducing trans-Golgi network-associated Lck signaling. Blood 119:786–797. doi:10.1182/blood-2011-08-373209. 22123847

[38] Nunn MF, Marsh JW. 1996. Human immunodeficiency virus type 1 Nef associates with a member of the p21-activated kinase family. J Virol 70:6157–6161. doi:10.1128/JVI.70.9.6157-6161.1996. 8709241 PMC190639

[39] Renkema GH, Manninen A, Mann DA, Harris M, Saksela K. 1999. Identification of the Nef-associated kinase as p21-activated kinase 2. Curr Biol 9:1407–1411. doi:10.1016/s0960-9822(00)80086-x. 10607567

[40] Rane CK, Minden A. 2014. P21 activated kinases: structure, regulation, and functions. Small GTPases 5:e28003. doi:10.4161/sgtp.28003. 24658305 PMC4160339

[41] Kirchhoff F, Schindler M, Bailer N, Renkema GH, Saksela K, Knoop V, Muller-Trutwin MC, Santiago ML, Bibollet-Ruche F, Dittmar MT, Heeney JL, Hahn BH, Munch J. 2004. Nef proteins from simian immunodeficiency virus-infected chimpanzees interact with p21-activated kinase 2 and modulate cell surface expression of various human receptors. J Virol 78:6864–6874. doi:10.1128/JVI.78.13.6864-6874.2004. 15194762 PMC421647

[42] Stolp B, Abraham L, Rudolph JM, Fackler OT. 2010. Lentiviral Nef proteins utilize PAK2-mediated deregulation of cofilin as a general strategy to interfere with actin remodeling. J Virol 84:3935–3948. doi:10.1128/JVI.02467-09. 20147394 PMC2849517

[43] Olivieri KC, Mukerji J, Gabuzda D. 2011. Nef-mediated enhancement of cellular activation and human immunodeficiency virus type 1 replication in primary T cells is dependent on association with p21-activated kinase 2. Retrovirology 8:64. doi:10.1186/1742-4690-8-64. 21819585 PMC3169461

[44] Kim S, Ikeuchi K, Byrn R, Groopman J, Baltimore D. 1989. Lack of a negative influence on viral growth by the nef gene of human immunodeficiency virus type 1. Proc Natl Acad Sci USA 86:9544–9548. doi:10.1073/pnas.86.23.9544. 2687883 PMC298533

[45] Wu Y, Olety B, Weiss ER, Popova E, Yamanaka H, Gottlinger H. 2019. Potent enhancement of HIV-1 replication by Nef in the absence of SERINC3 and SERINC5. mBio 10:e01071-19. doi:10.1128/mBio.01071-19. 31186327 PMC6561029

[46] Cheng H, Hoxie JP, Parks WP. 1999. The conserved core of human immunodeficiency virus type 1 Nef is essential for association with Lck and for enhanced viral replication in T-lymphocytes. Virology 264:5–15. doi:10.1006/viro.1999.9937. 10544125

[47] Witte V, Laffert B, Gintschel P, Krautkramer E, Blume K, Fackler OT, Baur AS. 2008. Induction of HIV transcription by Nef involves Lck activation and protein kinase C theta raft recruitment leading to activation of ERK1/2 but not NFκB. J Immunol 181:8425–8432. doi:10.4049/jimmunol.181.12.8425. 19050260

[48] Sleckman BP, Shin J, Igras VE, Collins TL, Strominger JL, Burakoff SJ. 1992. Disruption of the CD4-p56lck complex is required for rapid internalization of CD4. Proc Natl Acad Sci USA 89:7566–7570. doi:10.1073/pnas.89.16.7566. 1502168 PMC49751

[49] Sawai ET, Khan IH, Montbriand PM, Peterlin BM, Cheng-Mayer C, Luciw PA. 1996. Activation of PAK by HIV and SIV Nef: importance for AIDS in rhesus macaques. Curr Biol 6:1519–1527. doi:10.1016/S0960-9822(96)00757-9. 8939608

[50] Sawai ET, Baur A, Struble H, Peterlin BM, Levy JA, Cheng-Mayer C. 1994. Human immunodeficiency virus type 1 Nef associates with a cellular serine kinase in T lymphocytes. Proc Natl Acad Sci USA 91:1539–1543. doi:10.1073/pnas.91.4.1539. 8108442 PMC43195

[51] Arora VK, Molina RP, Foster JL, Blakemore JL, Chernoff J, Fredericksen BL, Garcia JV. 2000. Lentivirus Nef specifically activates Pak2. J Virol 74:11081–11087. doi:10.1128/jvi.74.23.11081-11087.2000. 11070003 PMC113188

[52] Wolf D, Witte V, Laffert B, Blume K, Stromer E, Trapp S, d'Aloja P, Schurmann A, Baur AS. 2001. HIV-1 Nef associated PAK and PI3-kinases stimulate Akt-independent Bad-phosphorylation to induce anti-apoptotic signals. Nat Med 7:1217–1224. doi:10.1038/nm1101-1217. 11689886

[53] Lu X, Wu X, Plemenitas A, Yu H, Sawai ET, Abo A, Peterlin BM. 1996. CDC42 and Rac1 are implicated in the activation of the Nef-associated kinase and replication of HIV-1. Curr Biol 6:1677–1684. doi:10.1016/s0960-9822(02)70792-6. 8994833

[54] Fackler OT, Murooka TT, Imle A, Mempel TR. 2014. Adding new dimensions: towards an integrative understanding of HIV-1 spread. Nat Rev Microbiol 12:563–574. doi:10.1038/nrmicro3309. 25029025 PMC5687059

[55] Fackler OT, Lu X, Frost JA, Geyer M, Jiang B, Luo W, Abo A, Alberts AS, Peterlin BM. 2000. p21-activated kinase 1 plays a critical role in cellular activation by Nef. Mol Cell Biol 20:2619–2627. doi:10.1128/MCB.20.7.2619-2627.2000. 10713183 PMC85477

[56] Nguyen DG, Wolff KC, Yin H, Caldwell JS, Kuhen KL. 2006. “UnPAKing” human immunodeficiency virus (HIV) replication: using small interfering RNA screening to identify novel cofactors and elucidate the role of group I PAKs in HIV infection. J Virol 80:130–137. doi:10.1128/JVI.80.1.130-137.2006. 16352537 PMC1317519

[57] Popov S, Popova E, Inoue M, Wu Y, Gottlinger H. 2018. HIV-1 gag recruits PACSIN2 to promote virus spreading. Proc Natl Acad Sci USA 115:7093–7098. doi:10.1073/pnas.1801849115. 29891700 PMC6142272

[58] Greenberg ME, Bronson S, Lock M, Neumann M, Pavlakis GN, Skowronski J. 1997. Co-localization of HIV-1 Nef with the AP-2 adaptor protein complex correlates with Nef-induced CD4 down-regulation. EMBO J 16:6964–6976. doi:10.1093/emboj/16.23.6964. 9384576 PMC1170300

[59] Jin YJ, Cai CY, Zhang X, Zhang HT, Hirst JA, Burakoff SJ. 2005. HIV Nef-mediated CD4 down-regulation is adaptor protein complex 2 dependent. J Immunol 175:3157–3164. doi:10.4049/jimmunol.175.5.3157. 16116206

[60] Gondim MV, Wiltzer-Bach L, Maurer B, Banning C, Arganaraz E, Schindler M. 2015. AP-2 is the crucial clathrin adaptor protein for CD4 downmodulation by HIV-1 Nef in infected primary CD4+ T cells. J Virol 89:12518–12524. doi:10.1128/JVI.01838-15. 26423947 PMC4665253

[61] Lindwasser OW, Smith WJ, Chaudhuri R, Yang P, Hurley JH, Bonifacino JS. 2008. A diacidic motif in human immunodeficiency virus type 1 Nef is a novel determinant of binding to AP-2. J Virol 82:1166–1174. doi:10.1128/JVI.01874-07. 18032517 PMC2224420

[62] Craig HM, Pandori MW, Guatelli JC. 1998. Interaction of HIV-1 Nef with the cellular dileucine-based sorting pathway is required for CD4 down-regulation and optimal viral infectivity. Proc Natl Acad Sci USA 95:11229–11234. doi:10.1073/pnas.95.19.11229. 9736718 PMC21624

[63] Bresnahan PA, Yonemoto W, Ferrell S, Williams-Herman D, Geleziunas R, Greene WC. 1998. A dileucine motif in HIV-1 Nef acts as an internalization signal for CD4 downregulation and binds the AP-1 clathrin adaptor. Curr Biol 8:1235–1238. doi:10.1016/s0960-9822(07)00517-9. 9811606

[64] Grzesiek S, Stahl SJ, Wingfield PT, Bax A. 1996. The CD4 determinant for downregulation by HIV-1 Nef directly binds to Nef. Mapping of the Nef binding surface by NMR. Biochemistry 35:10256–10261. doi:10.1021/bi9611164. 8756680

[65] Mangasarian A, Piguet V, Wang JK, Chen YL, Trono D. 1999. Nef-induced CD4 and major histocompatibility complex class I (MHC-I) down-regulation are governed by distinct determinants: N-terminal alpha helix and proline repeat of Nef selectively regulate MHC-I trafficking. J Virol 73:1964–1973. doi:10.1128/JVI.73.3.1964-1973.1999. 9971776 PMC104438

[66] Ren X, Park SY, Bonifacino JS, Hurley JH. 2014. How HIV-1 Nef hijacks the AP-2 clathrin adaptor to downregulate CD4. Elife 3:e01754. doi:10.7554/eLife.01754. 24473078 PMC3901399

[67] Jin YJ, Cai CY, Mezei M, Ohlmeyer M, Sanchez R, Burakoff SJ. 2013. Identification of a novel binding site between HIV type 1 Nef C-terminal flexible loop and AP2 required for Nef-mediated CD4 downregulation. AIDS Res Hum Retroviruses 29:725–731. doi:10.1089/AID.2012.0286. 23151229 PMC3903168

[68] Chen Y, Gershlick DC, Park SY, Bonifacino JS. 2017. Segregation in the Golgi complex precedes export of endolysosomal proteins in distinct transport carriers. J Cell Biol 216:4141–4151. doi:10.1083/jcb.201707172. 28978644 PMC5716290

[69] Rudolph JM, Eickel N, Haller C, Schindler M, Fackler OT. 2009. Inhibition of T-cell receptor-induced actin remodeling and relocalization of Lck are evolutionarily conserved activities of lentiviral Nef proteins. J Virol 83:11528–11539. doi:10.1128/JVI.01423-09. 19726522 PMC2772722

[70] Usmani SM, Murooka TT, Deruaz M, Koh WH, Sharaf RR, Di Pilato M, Power KA, Lopez P, Hnatiuk R, Vrbanac VD, Tager AM, Allen TM, Luster AD, Mempel TR. 2019. HIV-1 balances the fitness costs and benefits of disrupting the host cell actin cytoskeleton early after mucosal transmission. Cell Host Microbe 25:73–86. doi:10.1016/j.chom.2018.12.008. 30629922 PMC6456338

[71] Stolp B, Reichman-Fried M, Abraham L, Pan X, Giese SI, Hannemann S, Goulimari P, Raz E, Grosse R, Fackler OT. 2009. HIV-1 Nef interferes with host cell motility by deregulation of cofilin. Cell Host Microbe 6:174–186. doi:10.1016/j.chom.2009.06.004. 19683683

[72] Imle A, Abraham L, Tsopoulidis N, Hoflack B, Saksela K, Fackler OT. 2015. Association with PAK2 enables functional interactions of lentiviral Nef proteins with the exocyst complex. mBio 6:e01309-15. doi:10.1128/mBio.01309-15. 26350970 PMC4600113

[73] Mukerji J, Olivieri KC, Misra V, Agopian KA, Gabuzda D. 2012. Proteomic analysis of HIV-1 Nef cellular binding partners reveals a role for exocyst complex proteins in mediating enhancement of intercellular nanotube formation. Retrovirology 9:33. doi:10.1186/1742-4690-9-33. 22534017 PMC3382630

[74] Lundquist CA, Tobiume M, Zhou J, Unutmaz D, Aiken C. 2002. Nef-mediated downregulation of CD4 enhances human immunodeficiency virus type 1 replication in primary T lymphocytes. J Virol 76:4625–4633. doi:10.1128/jvi.76.9.4625-4633.2002. 11932428 PMC155097

[75] Roeth JF, Williams M, Kasper MR, Filzen TM, Collins KL. 2004. HIV-1 Nef disrupts MHC-I trafficking by recruiting AP-1 to the MHC-I cytoplasmic tail. J Cell Biol 167:903–913. doi:10.1083/jcb.200407031. 15569716 PMC2172469

[76] Noviello CM, Benichou S, Guatelli JC. 2008. Cooperative binding of the class I major histocompatibility complex cytoplasmic domain and human immunodeficiency virus type 1 Nef to the endosomal AP-1 complex via its mu subunit. J Virol 82:1249–1258. doi:10.1128/JVI.00660-07. 18057255 PMC2224416

[77] Craig HM, Reddy TR, Riggs NL, Dao PP, Guatelli JC. 2000. Interactions of HIV-1 nef with the mu subunits of adaptor protein complexes 1, 2, and 3: role of the dileucine-based sorting motif. Virology 271:9–17. doi:10.1006/viro.2000.0277. 10814565

[78] Lindwasser OW, Chaudhuri R, Bonifacino JS. 2007. Mechanisms of CD4 downregulation by the Nef and Vpu proteins of primate immunodeficiency viruses. Curr Mol Med 7:171–184. doi:10.2174/156652407780059177. 17346169

[79] Bedinger P, Moriarty A, von Borstel RC, II, Donovan NJ, Steimer KS, Littman DR. 1988. Internalization of the human immunodeficiency virus does not require the cytoplasmic domain of CD4. Nature 334:162–165. doi:10.1038/334162a0. 3260353

[80] Akagi T, Sasai K, Hanafusa H. 2003. Refractory nature of normal human diploid fibroblasts with respect to oncogene-mediated transformation. Proc Natl Acad Sci USA 100:13567–13572. doi:10.1073/pnas.1834876100. 14597713 PMC263854

[81] Ishikawa H, Meng F, Kondo N, Iwamoto A, Matsuda Z. 2012. Generation of a dual-functional split-reporter protein for monitoring membrane fusion using self-associating split GFP. Protein Eng Des Sel 25:813–820. doi:10.1093/protein/gzs051. 22942393

