# Supplementary figures and images for: AP-2 Adaptor Complex-Dependent Enhancement of HIV-1 Replication by Nef in the Absence of the Nef/AP-2 Targets SERINC5 and CD4
Source: mBio. 2023 Jan 9;14(1):e03382-22. doi: 10.1128/mbio.03382-22 (PMC9973267; doi:10.1128/mbio.03382-22)

Figure S1

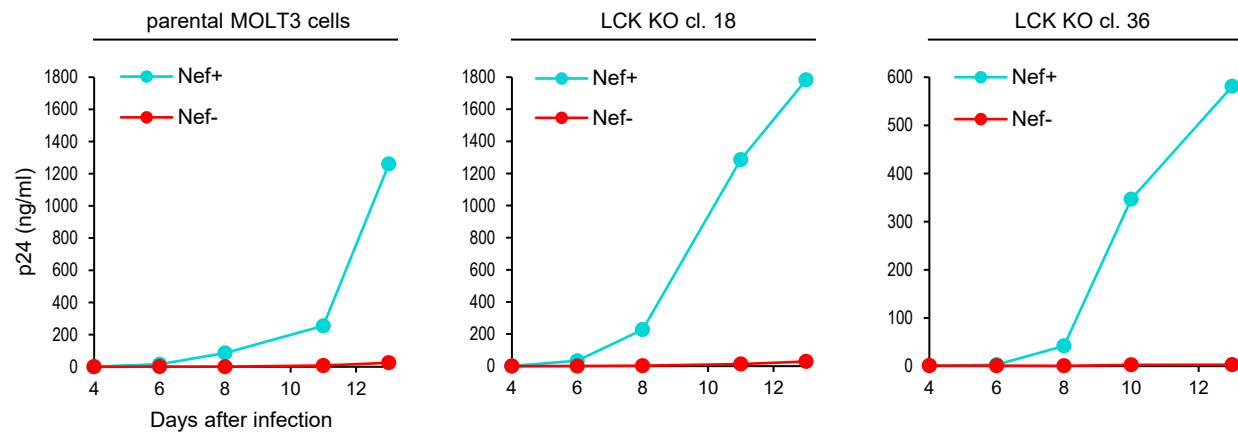

Supplement: FIG S1 [file mbio.03382-22-s0001.pdf]

Figure S2

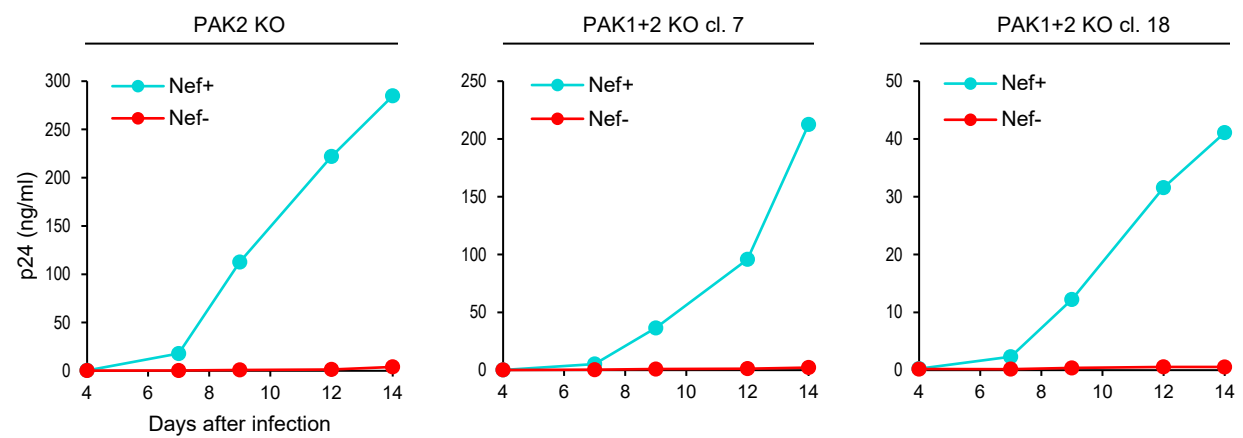

Supplement: FIG S2 [file mbio.03382-22-s0002.pdf]

Figure S3

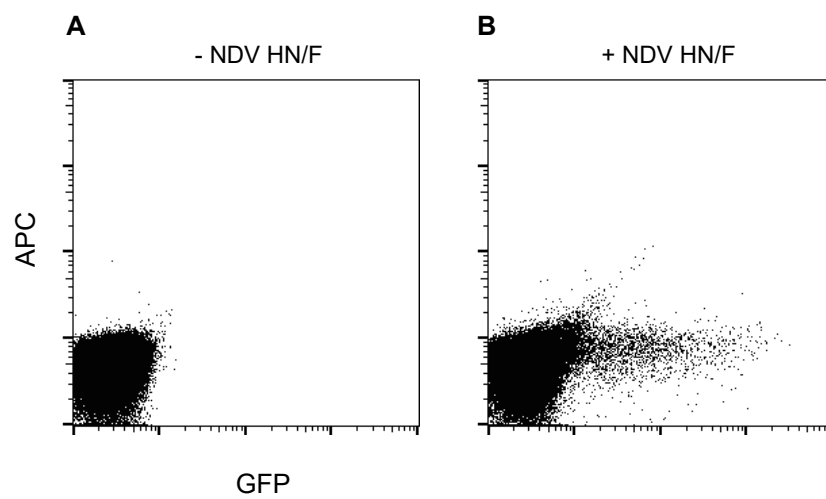

Supplement: FIG S3 [file mbio.03382-22-s0003.pdf]

Figure S4

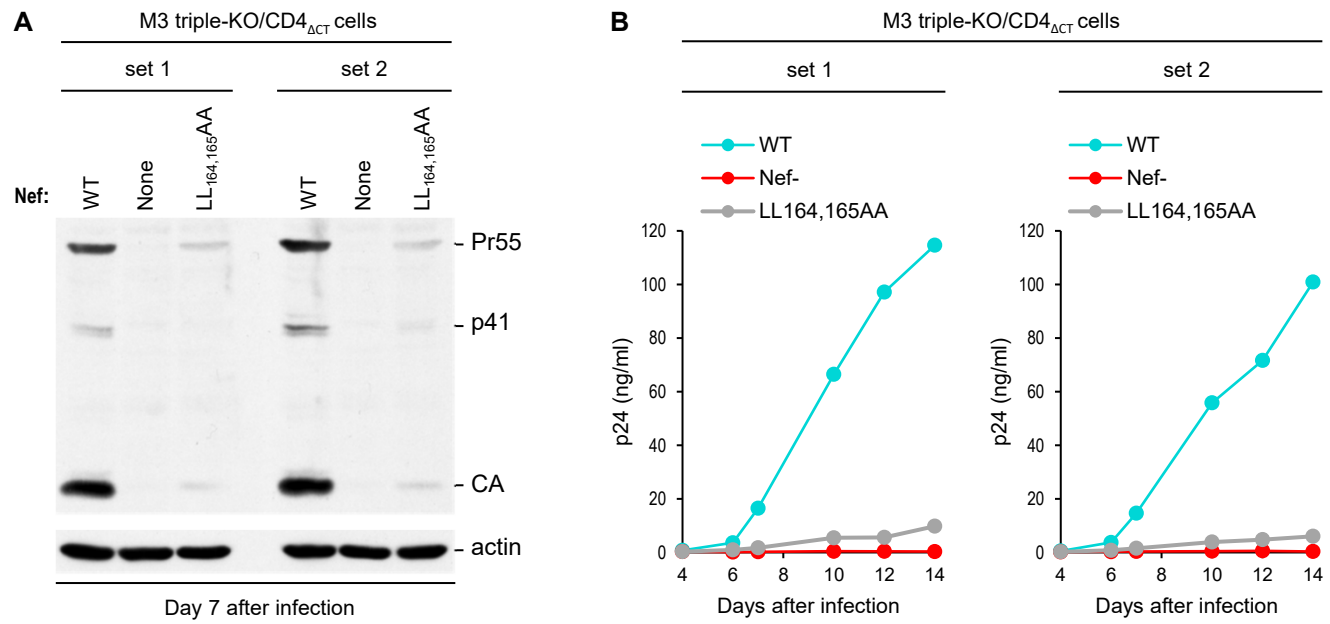

Supplement: FIG S4 [file mbio.03382-22-s0004.pdf]

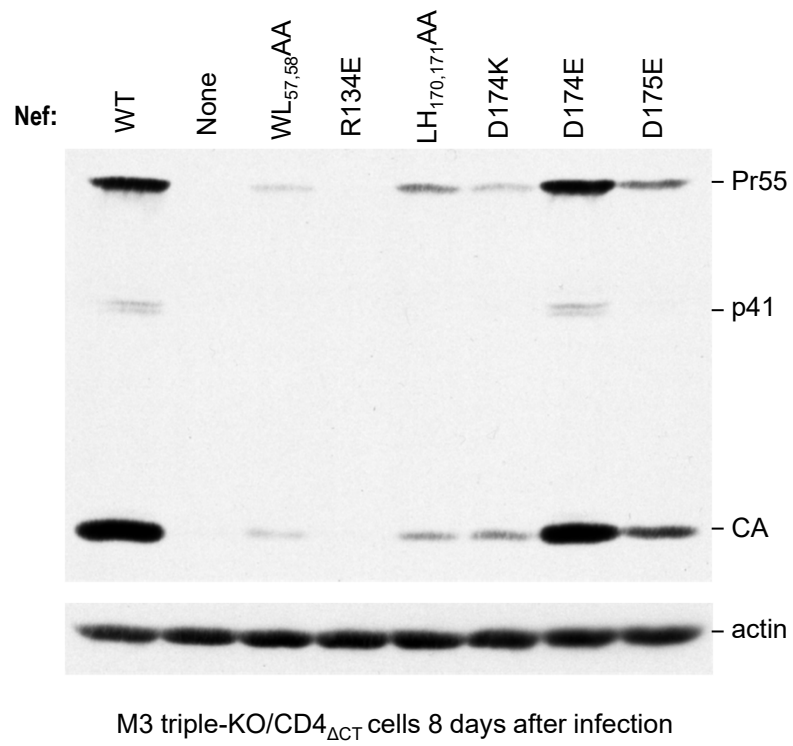

Supplement: FIG S5 [file mbio.03382-22-s0005.pdf]

Figure S6

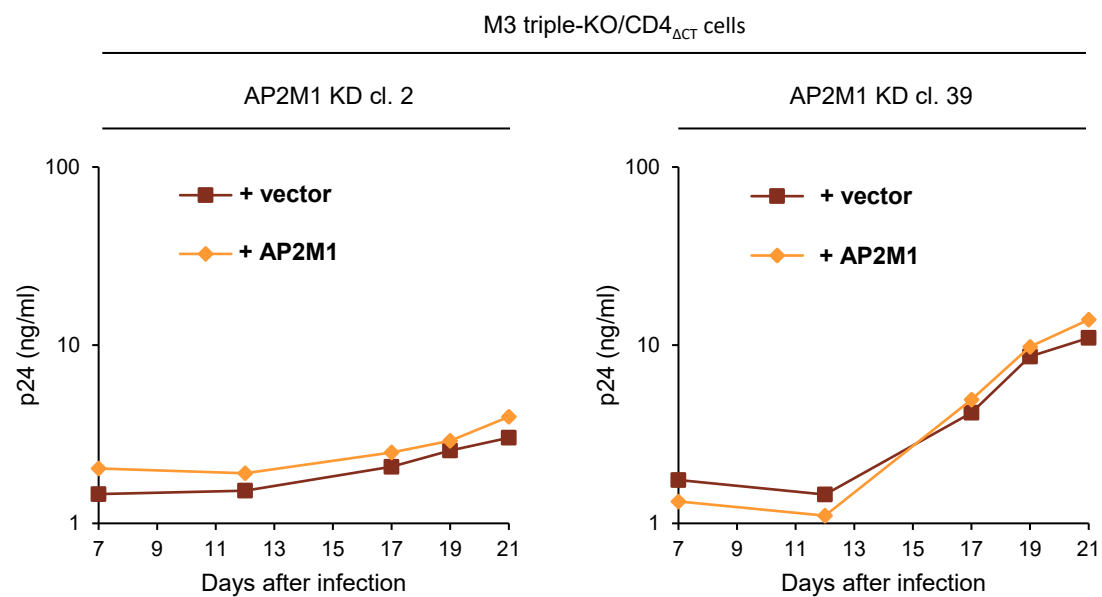

Supplement: FIG S6 [file mbio.03382-22-s0006.pdf]
